# Inflammatory biomarkers in pediatric PFAPA syndrome: a systematic review

**DOI:** 10.3389/fped.2026.1934055

**Published:** 2026-09-03

**Authors:** Paweł Treichel, Sylwia Kołtan, Jan Styczyński

**Affiliations:** 1 Department of Pediatrics, Hematology, Oncology, Immunology, and Transplantology, Nicolaus Copernicus University in Toruń, Ludwik Rydygier Collegium Medicum, Bydgoszcz, Poland; 2 Doctoral School of Medical and Health Sciences, Nicolaus Copernicus University in Toruń, Ludwik Rydygier Collegium Medicum, Bydgoszcz, Poland

**Keywords:** autoinflammatory disease, biomarkers, children, C-reactive protein, interferon-*γ*, PFAPA, serum amyloid A, systematic review

## Abstract

**Background:**

PFAPA (periodic fever, aphthous stomatitis, pharyngitis, cervical adenitis) is the most common periodic fever syndrome in childhood, yet remains a diagnosis of exclusion. No recent synthesis has quantitatively evaluated its circulating biomarkers. We aimed to assess the behavior of inflammatory biomarkers during febrile flares vs. asymptomatic intervals and evaluate their ability to distinguish PFAPA from acute infections and other auto inflammatory diseases.

**Methods:**

Following PRISMA 2020, PubMed and Scopus were searched for English-language studies (1 January 2018–07 June 2026) including children (0–18 years) with confirmed PFAPA reporting at least one of eleven predefined biomarkers (WBC, neutrophils, lymphocytes, CRP, SAA, serum calprotectin, IL-6, IL-1β, IL-18, TNF-α, IFN-*γ*).

**Results:**

Seven studies (710 children; five countries; two prospective, five retrospective; 2019–2026) were included from 170 records. CRP and SAA increased markedly during flares and normalized between episodes, with SAA showing the greatest dynamic range. However, neither CRP, WBC, neutrophils, nor IL-6 reliably distinguished PFAPA flares from acute infections. IL-1β was consistently undetectable in serum despite the inflammasome-driven pathophysiology. IFN-*γ* emerged as the only consistently discriminating biomarker, elevated in PFAPA compared with infections across three independent cohorts. In one cohort, the IFN-*γ*/IL-6 ratio (AUC 0.79) and a composite model including IL-10 and platelet count (AUC 0.95) demonstrated promising diagnostic performance. Both cut-offs were derived and tested in the same at-risk-of-bias cohort without external validation and should be read as derivation-stage estimates. No biomarker differentiated PFAPA from other auto inflammatory diseases: the only direct comparison (with SURF) showed overlapping values without comprehensive statistical testing, and differentiation from familial Mediterranean fever relied on clinical criteria. For discrimination outcomes, GRADE certainty did not exceed low; CRP as an activity marker reached moderate-to-high certainty.

**Conclusions:**

CRP and SAA are reliable markers of PFAPA activity but lack discriminating value vs. infection. IFN-*γ*, particularly in combination with IL-6, is the most promising candidate for differentiation, though current evidence is limited and may be biased. No circulating biomarker distinguishes PFAPA from other autoinflammatory diseases, representing a major unmet need. PFAPA, therefore, remains a clinical diagnosis requiring exclusion of monogenic syndromes.

## 1 Introduction

Periodic fever, aphthous stomatitis, pharyngitis, and cervical adenitis (PFAPA) syndrome is the most common periodic fever syndrome of childhood (1–3). Its clinical tetrad defines it, regularly recurring fevers accompanied by aphthous stomatitis, pharyngitis or tonsillitis, and/or cervical adenitis, with complete recovery between episodes and normal growth and development (4, 5). Onset is typically before five years of age, the reported incidence is approximately 2.3 per 10,000 children under five with a slight male predominance, and the disorder usually resolves spontaneously by early adolescence (6, 7). Although benign and self-limiting, it imposes a considerable burden on affected children and their families through repeated febrile episodes, missed school, and recurrent, often unnecessary, medical care (8, 9).

PFAPA has no confirmatory laboratory or genetic test; the diagnosis remains clinical and is made by exclusion of infectious, immunodeficient, neoplastic, and monogenic autoinflammatory causes of recurrent fever (3, 10). Several partly overlapping criteria sets are in use. The original description by Marshall et al. (1987), the widely applied criteria of Thomas et al. (1999), the modified Padeh criteria, and the Eurofever/PRINTO classification, with further criteria proposed for adult-onset disease (4, 5, 11–13). The first PFAPA classification, described by Marshall, is presented in Table 1.

**Table 1:** PFAPA diagnostic criteria based on marshall et al. (4).

Onset of the disease in early childhood, usually by the age of 5
Regular recurring episodes of fever, lasting approximately 5 days, with co-occurring, persistent symptoms and a minimum of two of the following: a) Aphthous stomatitis and/or pharyngitis (with or without cervical lymph nodes) in the absence of other signs of respiratory infection b) markers of inflammation such as leukocytosis or elevated erythrocyte sedimentation rate
Completely asymptomatic period between episodes (usually less than 10 weeks), mild long-term course, normal growth parameters, clear absence of sequelae
Exclusion of cyclic neutropenia
Exclusion of other causes of recurrent fever (Familial Mediterranean Fever, Familial Hibernian Fever, hyper-IgD syndrome, Behcet disease) based on family history and absence of typical clinical signs and laboratory markers
Absence of clinical and laboratory markers of immune deficiencies, autoimmune diseases, and chronic infections

Because none of these definitions constitutes a reference standard, their coexistence contributes to diagnostic uncertainty and to the well-recognized problems of delayed and incorrect diagnosis (10, 14).

Mechanistically, PFAPA is regarded as a polygenic autoinflammatory disorder driven by dysregulated innate immunity rather than by autoantibodies or antigen-specific adaptive responses (15–17). Febrile episodes are characterized by inflammasome activation and overproduction of interleukin-1β by monocytes, with downstream neutrophil recruitment, complement activation, and a T-helper-1/interferon-*γ* signature. Familial clustering consistent with incomplete-penetrance inheritance is also well documented (18–23). This innate immune activation provides the rationale for measuring circulating inflammatory mediators during flares and underlies the expectation that these mediators might assist in diagnosis. Corticosteroids reliably abort individual attacks, and tonsillectomy can be curative, but these therapeutic responses do not resolve the diagnostic question (2, 8, 24).

The central diagnostic challenge is differentiation. During a flare, PFAPA closely mimics common childhood infections. Most often, PFAPA simulates streptococcal pharyngitis or tonsillitis, and clinically overlaps with other autoinflammatory diseases, including the monogenic periodic fevers (familial Mediterranean fever, mevalonate kinase deficiency, tumor-necrosis-factor-receptor-associated periodic syndrome, and cryopyrin-associated periodic syndromes) and the increasingly recognized syndrome of undifferentiated recurrent fever (SURF), which itself retains an interleukin-1 signature (25–27). Failure to distinguish PFAPA from infection leads to unnecessary antibiotic courses, whereas failure to distinguish it from monogenic disease can delay disease-specific treatment; both consequences make an objective discriminating marker clinically valuable (9, 10, 14).

A range of inflammatory biomarkers rises during PFAPA flares, including the acute-phase reactants C-reactive protein and serum amyloid A, serum calprotectin, and several pro-inflammatory cytokines (28–30). The recurring difficulty is that these markers are sensitive indicators of inflammation but not disease-specific: comparable elevations occur in bacterial infections and other autoinflammatory syndromes, so an isolated elevated value rarely identifies PFAPA. Reported studies have also differed widely in design, comparator group, sampling time point (flare vs. interval), and assay units, which has hampered direct comparison. Whether any single biomarker, ratio, or composite can reliably distinguish PFAPA from its infectious and autoinflammatory mimics in children, therefore, remains unclear and has not, to our knowledge, been systematically appraised.

We therefore conducted a systematic review of inflammatory biomarkers in children with PFAPA syndrome. Framed by a PECO question: population, children with PFAPA; exposure, circulating inflammatory biomarkers; comparators, infection, other autoinflammatory diseases, or the asymptomatic interval; outcome, discriminating performance, the review aimed to identify which biomarkers have been studied. We would also like to synthesize reported biomarker values at flare and between episodes, and to evaluate how well they separate PFAPA from infection and from other autoinflammatory diseases, thereby clarifying whether any biomarker currently approaches clinical usefulness and where the principal evidence gaps lie.

No systematic review published since 2018 has comprehensively synthesized quantitative biomarker data (WBC, neutrophils, lymphocytes, CRP, SAA, serum calprotectin, IL-6, IL-1β, IL-18, TNF-α, IFN-*γ*) across the full spectrum of available pediatric PFAPA studies. This gap is clinically relevant: accurate biomarker characterization could accelerate diagnosis, reduce antibiotic overuse, and stratify patients for targeted anti-cytokine therapy.

## 2 Materials and methods

### 2.1 Study design

The primary aim of this systematic review was to comprehensively synthesize and critically appraise quantitative evidence on inflammatory biomarker profiles in pediatric patients (0–18 years) with confirmed PFAPA syndrome, based on studies published between 2018 and 2026 with accessible full texts.

Particular emphasis was placed on the analysis of individual biomarkers during febrile episodes and asymptomatic inter-flare periods, as well as on evaluating their potential diagnostic utility, including their ability to discriminate PFAPA from other febrile conditions in the acute clinical setting.

### 2.2 Protocol and registration

**Figure 1:**
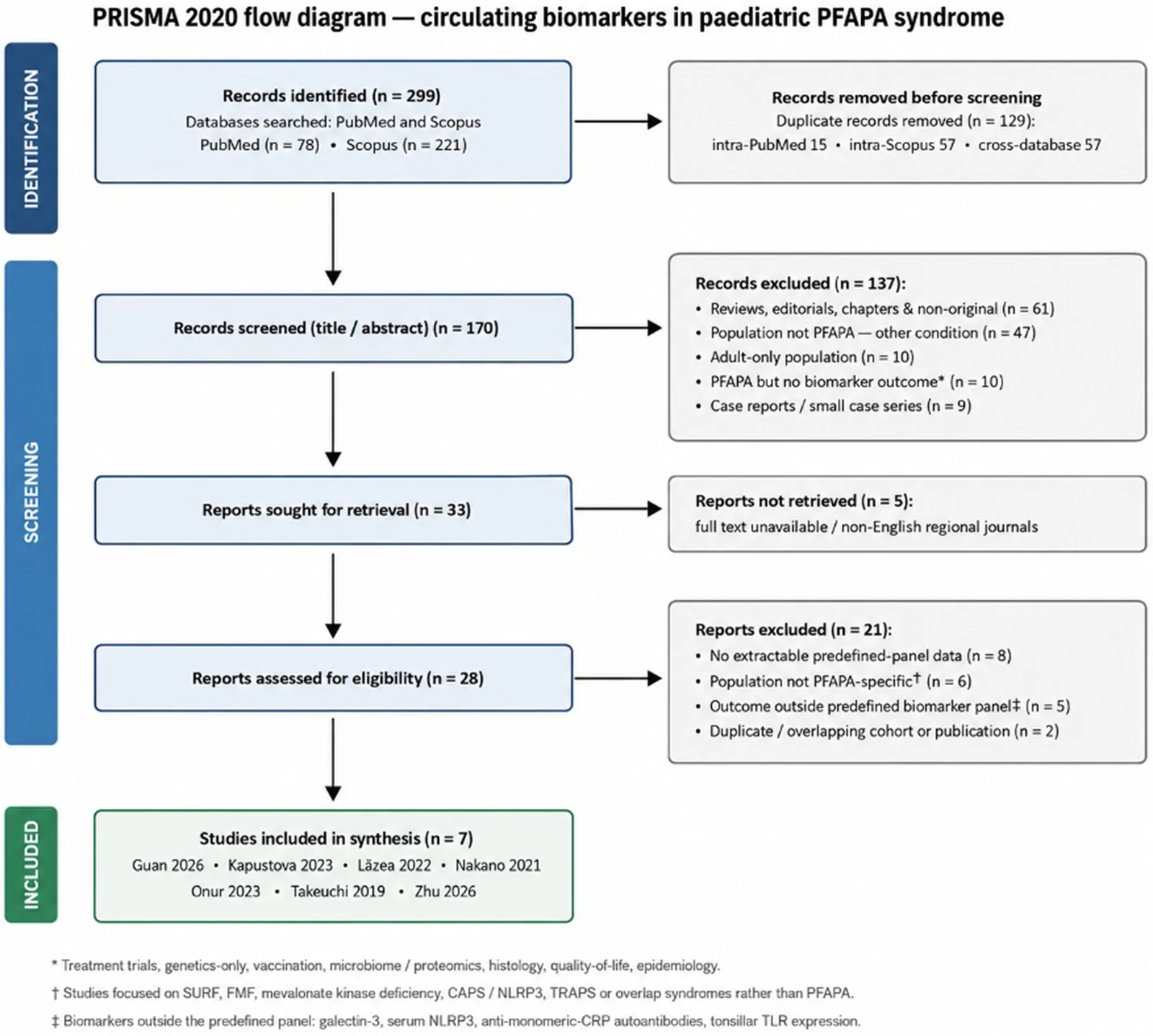
PRISMA 2020 flow diagram.

This systematic review was conducted in accordance with Preferred Reporting Items for Systematic Reviews and Meta-Analyses (PRISMA 2020) reporting guidelines. A completed PRISMA checklist is included in the Supplementary Materials (Supplementary Table 1), while the PRISMA flow diagram is presented in Figure 1. The review was not prospectively registered in PROSPERO. We acknowledge this as a limitation. The protocol (eligibility, extraction, and analysis plan) was nonetheless finalized before data extraction and is provided in Supplementary File S1.

### 2.3 Eligibility criteria

The following criteria served as the basis for the eligibility assessment and subsequent inclusion or exclusion of studies.

#### 2.3.1 Inclusion criteria

- Original clinical studies (prospective or retrospective cohorts, cross-sectional studies, case-control designs, or randomized controlled trials)
- Pediatric population (age 0–18 years) with confirmed PFAPA syndrome according to any validated diagnostic criteria (Thomas/Marshall 1999, Gattorno/Eurofever/PRINTO 2019, Takeuchi 2019, or equivalent)
- Reporting quantitative or semi-quantitative data for at least one of the following biomarkers: WBC, neutrophils, lymphocytes, CRP, SAA, serum calprotectin, IL-6, IL-1β, IL-18, TNF-α, or IFN-*γ*
- Published in English between 1 January 2018 and 07 June 2026
- Full text available

#### 2.3.2 Exclusion criteria

- Review articles, meta-analyses, editorials, commentaries, and letters
- Case reports and case series with fewer than 5 PFAPA patients
- Studies with exclusively adult populations (age >18 years)
- Animal studies
- Studies in which PFAPA served only as a reference/comparison group, with no PFAPA-specific biomarker values reported.
- Conference abstracts without corresponding full-text publication
- Studies without extractable biomarker data for PFAPA patients

### 2.4 Information sources and search strategy

A systematic literature search was conducted in two bibliographic databases, PubMed and Scopus, each interrogated with three complementary query blocks combining the disease term (PFAPA and its expansions) with biomarker, laboratory, and differential-diagnosis terms; the complete search strategies for both databases are provided in the accompanying search-strategy file (Supplementary Table 2).

These two databases were selected because together they index the vast majority of pediatric rheumatology and immunology literature in which PFAPA biomarker studies are published. This restriction is acknowledged as a limitation of the search strategy (Section 4): studies indexed only in Embase, Web of Science, or the Cochrane Library, or reported in grey literature, may not have been identified.

Searches were performed using English-language terms and restricted to articles published between 1 January 2018 and 07 June 2026. No automated database filters were applied during the search process. The final search was performed on 07 June 2026. Two independent reviewers screened titles and abstracts against predefined eligibility criteria; the remaining reports underwent full-text assessment, including relevant supplementary files [as in Guan et al. (31)]. No additional searches of reference lists, citation tracking in Google Scholar or Web of Science, or grey literature sources were conducted.

### 2.5 Study selection

Three complementary search strings executed in PubMed/MEDLINE (National Library of Medicine, USA) and Scopus on 07 June 2026 yielded 299 records (S1 = 37, S2 = 18, S3 = 23 in PubMed; S1 = 95, S2 = 64, S3 = 62 in Scopus). S1–S3 refer to predefined keyword-based search strategies detailed in Supplementary Table 1.

De-duplication was performed using a normalized Digital Object Identifier (lower-cased, prefix-stripped), with a fallback to a normalized title key (lower-cased, punctuation removed, whitespace collapsed) for the small number of records lacking a DOI. Duplicates were removed in two passes. First, within each database across its three queries, then across the two databases, yielding a single transparent count of records removed before screening. The deduplication process, as well as the subsequent screening and analysis, was conducted manually and automatically using the Rayyan platform (a web-based software for systematic review screening) by two independent reviewers (32). Disagreements were resolved by consensus.

After removing 129 duplicates, 170 unique records were obtained from database searches. Following title/abstract screening, 137 records were excluded (review articles, editorials, chapters, non-original *n* = 61; population not PFAPA *n* = 47; adults-only *n* = 10; no biomarker outcome *n* = 10; case reports/small case series *n* = 9). Thirty-three reports were sought for retrieval, of which 5 were excluded because of the unavailability of full text or non-English regional journals. Twenty-eight full-texts were analyzed for eligibility, and twenty-one reports were excluded (no extractable predefined-panel data *n* = 8; population not PFAPA-specific *n* = 6; outcome outside predefined biomarker panel *n* = 5; duplicate/overlapping cohort or publication *n* = 2). Seven studies from database searches were included.

Two independent reviewers screened and analyzed titles and abstracts against eligibility criteria. Full texts were retrieved and assessed for all records that passed the initial screening. Disagreements were resolved by consensus by a third reviewer. The selection process is documented in the PRISMA 2020 flow diagram (Figure 1).

Nakano et al. (33) contributed only three PFAPA patients, but met the review's eligibility criteria: the pre-specified small-sample exclusion applied to case reports and case series, not to controlled designs. Nakano et al. is a retrospective comparative study with a defined comparator (recurrent tonsillitis) and formal between-group testing, so it falls outside that exclusion as written rather than by *post-hoc* exception. In line with GRADE and Cochrane practice, its small sample was handled through transparent downweighting rather than exclusion. The high risk of bias (Supplementary File S2) and a hypothesis-generating rating contributed to the GRADE downgrading (Supplementary File S3). No conclusion depends on it: its key signal, elevated interferon-*γ* in PFAPA vs. infection, is independently reproduced in two larger cohorts [Zhu et al. (34) and Takeuchi et al. (35)].

Studies measuring tissue compartments (e.g., tonsillar) rather than circulating biomarkers were excluded as outside the predefined exposure [e.g., Luu et al. (27)].

Non-peer-reviewed preprints and records not indexed in PubMed or Scopus were excluded from the search. They were not eligible [e.g., Gołuchowska et al. (36), a Research Square preprint reporting only single-group descriptive laboratory values without a flare-vs.-interval, infection, or autoinflammatory comparator].

### 2.6 Data extraction

A standardized extraction form was applied to each included study. For every study the following were extracted by two reviewers independently: first author and year; the digital object identifier and, where available, the PubMed identifier; country of origin; study design; the number of patients with PFAPA and in the comparator groups; the diagnostic criteria applied; the mean or median patient age; the disease phase assessed (febrile attack, asymptomatic interval, or both), together with the comparator group where one was used (i.e., acute infection, SURF or other); and the numerical values, mean ± standard deviation or median with interquartile range, as originally published, for each of the eleven predefined target biomarkers (WBC, NEUT, LYMPH, CRP, SAA, calprotectin, IL-6, IL-1β, IL-18, TNF-α and IFN-*γ*).

Where a value was not available from the abstract, the full text was accessed via PMC or the publisher record and, where relevant, the supplementary files [as for the immune indices of Guan et al.’s supplementary Tables (31)]. When a specific numerical value remained unavailable despite full-text and supplementary access, the entry was coded as “Not reported” (NR).

### 2.7 Risk of bias assessment

The methodological quality of the included studies was appraised using the Joanna Briggs Institute (JBI) Critical Appraisal Checklist for Analytical Cross-Sectional Studies, applied uniformly to all seven cohorts so that heterogeneous observational designs could be assessed on a common set of domains; for the two studies built on paired within-patient flare-vs.-interval sampling, the equivalent JBI cohort items were additionally considered when interpreting the confounding domains. Two reviewers (P.T.; S.K.) independently assessed each study for potential selection, diagnostic, measurement, and reporting bias, and any disagreements were resolved through discussion and consensus or, where necessary, by a third reviewer (J.S.).

The JBI checklist was applied uniformly across all seven cohorts to appraise heterogeneous observational designs using a common set of domains. Because the JBI cross-sectional tool does not capture the patient-selection, index-test, reference-standard, and flow-and-timing domains specific to diagnostic test-accuracy research, the three studies that additionally reported diagnostic-accuracy estimates (Onur & Onur, Zhu et al., and Takeuchi et al.) were also appraised with the QUADAS-2 tool (37) across its four domains, each rated for risk of bias and, for the first three, for applicability concerns. QUADAS-2 was not extended to the remaining four studies, which reported only within-group (flare-vs.-interval) or descriptive between-group comparisons without accuracy estimates. On QUADAS-2, all three accuracy studies were judged at high risk of bias in the patient-selection and index-test domains, reflecting two-gate (case–control–type) sampling and in-sample derivation of discriminating thresholds. Their ROC/AUC estimates should be regarded as derivation-stage rather than externally validated performance. The full QUADAS-2 appraisal is provided in Supplementary File S4.

The full item-level JBI appraisal, the overall risk-of-bias judgments, and the JBI traffic-light summary are presented in Supplementary File S2 and Supplementary Table 3; the QUADAS-2 appraisal of the three diagnostic-accuracy studies, with its traffic-light summary, is presented in Supplementary File S4.

### 2.8 Data synthesis and certainty assessment (GRADE)

Because of substantial heterogeneity in comparator groups, assay platforms, units of measurement, and reporting formats, a quantitative meta-analysis was judged inappropriate, and a structured qualitative (narrative) synthesis was undertaken instead. Data were extracted into structured summary tables (study characteristics, hematological parameters, inflammatory markers, and cytokines, and additional acute-phase, cellular, and chemokine markers), with each study treated as an independent unit and no aggregation of values across studies. Continuous data are reported as originally published (mean ± standard deviation, median with interquartile or full range), and missing parameters are marked “Not reported”; where a study's biomarker values resided in supplementary files [i.e., in Guan et al.’s supplementary Tables (31)], the supplementary Table was obtained and its data incorporated into the analysis.

We performed a structured GRADE appraisal across the principal questions of this review: the change in each biomarker during the febrile attack vs. the asymptomatic interval (disease-activity monitoring), the discrimination of PFAPA from acute infection, and the discrimination of PFAPA from other autoinflammatory diseases. The certainty of evidence was rated for each of the eleven predefined biomarkers using the standard GRADE domains (risk of bias, inconsistency, indirectness, imprecision, and publication bias), starting at low certainty given the observational nature of the included studies and allowing possible upgrading for large and consistent effects. Certainty was formally rated for the activity and infection-discrimination questions, whereas differentiation of PFAPA from other autoinflammatory diseases was assessed narratively because too few studies addressed it. The summary is presented in Supplementary File S3.

For the discrimination outcomes (Question B), the risk-of-bias domain was additionally informed by the QUADAS-2 appraisal of the three diagnostic-accuracy studies (Supplementary File S4); the high risk of bias identified there was carried through as a risk-of-bias downgrade for the interferon-*γ* discrimination outcome.

## 3 Results

This systematic review included seven studies examining circulating biomarkers in children with PFAPA syndrome. The study-selection process is summarized in the PRISMA 2020 flow diagram (Figure 1). In total, 299 records were identified across PubMed (*n* = 78) and Scopus (*n* = 221), and 170 remained after 129 duplicates were removed. Most title-and-abstract exclusions (*n* = 137) concerned non-original publications (reviews, editorials, and book chapters), studies of other autoinflammatory or unrelated conditions, adult-only populations, and papers without an extractable biomarker outcome. Of the 33 reports sought, 5 could not be obtained. Twenty-one of the 28 full texts assessed were excluded, most often because the analytes measured fell outside the predefined panel, the population was not PFAPA-specific, or the predefined biomarkers could not be extracted in a comparative format.

Ultimately, seven studies fulfilled the inclusion criteria. These comprised two prospective studies and five retrospective cohorts, with comparisons spanning the febrile flare vs. the asymptomatic interval, PFAPA vs. acute infection, and PFAPA vs. other auto inflammatory disease (SURF, familial Mediterranean fever) or healthy controls, reflecting both activity-monitoring and differential-diagnostic uses of circulating biomarkers. Data from all included studies were compiled into structured summary tables to facilitate comparison of study characteristics, methodologies, and key findings: study characteristics (Table 2), hematological parameters (Table 3), acute-phase reactants (Table 4), cytokines and interferons (Table 5), and additional or derived markers (Table 6). The certainty of evidence for each biomarker is summarized in the GRADE evidence profile (Supplementary File S3), and the per-study risk-of-bias appraisal is presented in Supplementary File S2 and Supplementary Table 3.

**Table 2:** Characteristics of included studies.

Study, Year	PMID/DOI	Country	N (PFAPA)	Age	Design	Phase	COMPARED TO	Diagnostics Criteria
Takeuchi et al. (2019) (35)	31131563 10.1111/1756-185X.13610	Japan	257	Mean (onset): 2.7 ± 1.6 years	Retrospective cohort study	Flare/Inter-flare (referred to as Attack-free periods)	Healthy adult controls (*n* = 12), bacterial infections (*n* = 104), and PFAPA inter-flare phase (intra-patient comparison)	Marshall and Thomas criteria + own criteria (same cohort, criterion contamination)
Nakano et al. (2021) (33)	33994477 10.2152/jmi.68.38	Japan	3	Mean: 5.2 ± 0.2 years	Retrospective observational study (chart review)	Flare (referred to as Febrile episodes)	Recurrent tonsillitis (*n* = 4)	Thomas, Padeh, and Gattorno criteria
Lazea et al. (2022) (38)	36061962 10.2147/IJGM.S373942	Romania	39	Median (onset): 1.58 years	Retrospective, observational cohort study	Flare/Inter-flare (referred to as Inter-episode)	PFAPA inter-flare phase (intra-patient comparison)	Thomas criteria
Onur et al. (2023) (39)	37428978 10.1002/jcla.24934	Turkey	67	Median 43 months	Prospective observational study	Flare (referred to as Flare-ups)	Streptococcal pharyngitis (*n* = 74)	Marshall and Thomas criteria
Kapustova et al. (2023) (40)	38143757 10.3389/fimmu.2023.1302875	Slovakia	142	Mean: 6.81 ± 3.03 years (onset: 2.31 ± 2.02)	Prospective clinical study	Flare/Inter-flare (referred to as Attack-free period)	PFAPA inter-flare phase (intra-patient comparison)	Thomas criteria
Guan et al. (2026) (31)	41972166 10.3389/fimmu.2026.1791904	China	135	Median (onset): 1.50 years	Retrospective cohort study	Flare/Inter-flare (referred to as Acute attacks/Remission period)	PFAPA inter-flare phase, SURF-periodic flare (*n* = 38), and SURF non-periodic non-flare (*n* = 34)	Eurofever/PRINTO + Marshall
Zhu et al. (2026) (34)	41766872 10.3389/fimmu.2026.1683221	China	67	Median (onset): 4.7 years	Retrospective single-center study	Flare (referred to as Febrile episodes)	Bacterial infections (*n* = 160)	Eurofever/PRINTO classification and CARRA PFAPA work group

Footnotes: ^1^ Takeuchi 2019: The authors developed and validated their own new diagnostic criteria using the same cohort of patients, which constitutes a criterion contamination bias. ^2^ Nakano 2021: Extremely small sample size (*N* = 3 for PFAPA, *N* = 4 for recurrent tonsillitis), which drastically limits the statistical power and generalizability of the cytokine findings. ^3^ Lazea 2022: Retrospective design with no genetic testing performed to rule out monogenic periodic fever syndromes, relying solely on clinical criteria, which introduces a high risk of misclassification bias. ⁴ Kapustova 2023: This is primarily a prospective clinical study investigating ketotifen prophylaxis; the biomarker comparison (flare vs. inter-flare) is a secondary objective, and it lacks a healthy control group. Inter-flare samples were collected at least two weeks after the attack. ⁵ Onur & Onur 2023: Lacks a healthy control group, solely comparing PFAPA to streptococcal tonsillitis. Furthermore, it did not evaluate the clinical findings of PFAPA (relying solely on the diagnosis) or assess specific cytokine profiles. ⁶ Guan 2026: This is a 10-year retrospective epidemiology study of pediatric Fever of Unknown Origin (FUO), where PFAPA is one of several diagnoses. Biomarker data for PFAPA are not always reported separately in a dedicated table but are instead aggregated with other autoinflammatory conditions. ⁷ Zhu 2026: Significant time-to-sampling bias: the median time from fever onset to sampling was significantly shorter for PFAPA (2 days) compared to bacterial infections (6 days), which may artificially inflate differences in cytokines with short half-lives (e.g., IFN-*γ*). Additionally, no inter-flare baseline samples were collected.; SURF, syndrome of undifferentiated recurrent fever.

**Table 3:** Hematological parameters (abridged; full version in Supplementary File S5, Supplementary Table 5a).

Study, Year	Contrast	WBC (flare/comp.)	Neutrophils (flare/comp.)	Lymphocytes (flare/comp.)	Key result
Zhu et al., 2026 (34)	PFAPA vs. bacterial	10.7/11.6 × 10⁹/L	7.5/7.2 × 10⁹/L	NR	WBC *p* = 0.477, NEUT *p* = 0.835 - NS
Guan et al., 2026 (31)	PFAPA flare vs. remission	12.35/7.13 × 10⁹/L	8.46/3.00 × 10⁹/L	2.61/3.38 × 10⁹/L	↑WBC, ↑NEUT, ↓LYMPH at flare; SURF subgroups near-identical at flare (full data: Table S5a)
Kapustova et al., 2023 (40)	PFAPA flare vs. interval	11.95/8.05 × 10⁹/L	6.45/3.15 × 10⁹/L	2.75/3.82 × 10⁹/L	↑WBC, ↑NEUT (*p* < 0.0001); ↓LYMPH (*p* = 0.0009)
Onur & Onur, 2023 (39)	PFAPA vs. strep	14,040/15,130/mm^3^	8,820/9,500/mm^3^	2,860/2,760/mm^3^	WBC *p* = 0.231, NEUT *p* = 0.233, LYMPH *p* = 0.170 — NS
Lazea et al., 2022 (38)	PFAPA flare vs. inter-episode	14,839/8,922^a^/mm^3^	9,977/3,386/mm^3^	3,683/4,568/mm^3^	↑WBC (*p* < 0.0001), ↑NEUT (*p* = 0.0001); ↓LYMPH NS (*p* = 0.0707)
Nakano et al., 2021 (33)	PFAPA vs. recurrent tonsillitis	10,977/13,015/mm^3^	NR	NR	WBC NS
Takeuchi et al., 2019 (35)	PFAPA flare only	12,500/µL	66.1% Seg + 3.7% Band (%, not absolute)	NR	Marked leucocytosis; neutrophils as % only; no flare/remission contrast

Band, band neutrophils; comp., comparator (interval or comparator disease per the Contrast column); LYMPH, lymphocytes; NEUT, neutrophils; NR, not reported; NS, not significant; PFAPA, periodic fever, aphthous stomatitis, pharyngitis, and adenitis; SD, Standard Deviation; Seg, segmental neutrophils; SURF, syndrome of undifferentiated recurrent fever; WBC, white blood cells; ×10⁹/L, times ten to the ninth per liter;/mm^3^, per cubic millimeter;/µL, per microliter.

a Upper inter-episode bound (138,000/mm^3^) is a probable source typo, reported verbatim. Units differ (×10⁹/L vs./mm^3^ vs./µL). Full between-group comparisons for all groups: Supplementary File S5, Supplementary Table 5a.

**Table 4:** Acute phase reactants (CRP, SAA, serum calprotectin) and proteins (abridged; full version in Supplementary File S5, Supplementary Table 5b).

Study, Year	Contrast	CRP (flare/comp.)	SAA (flare/comp.)	Calprotectin (flare/interval)	Key result
Zhu et al., 2026 (34)	PFAPA vs. bacterial	41.1/49.4 mg/L	113.2/116.5 mg/L	NR	CRP & SAA NS
Guan et al., 2026 (31)	PFAPA flare (many comparator groups—see: Supplementary File S5)	50.54 mg/L	238.14 mg/L	NR	PFAPA CRP [mg/L] > bacterial 26.56, viral 23.45, mycoplasma 35.86; SAA [mg/L] highest in mycoplasma 303.78 and SURF-periodic 320.98 (full data: Supplementary Table 5b)
Kapustova et al., 2023 (40)	PFAPA flare vs. interval	50/0.95 mg/L	132/4 mg/L	3.64/5.92 µg/mL	↑↑CRP, ↑↑SAA (*p* < 0.0001); calprotectin HIGHER in interval (*p* = 0.036)
Onur & Onur, 2023 (39)	PFAPA vs. strep	32/27.13 mg/dL^1^	NR	NR	↑CRP (*p* = 0.026)
Lazea et al., 2022 (38)	PFAPA flare vs. inter-episode	7.9/0.5 mg/dL	NR	NR	↑CRP (*p* < 0.0001)
Nakano et al., 2021 (33)	PFAPA vs. recurrent tonsillitis	3.13/4.48 mg/dL	NR	NR	CRP NS
Takeuchi et al., 2019 (35)	PFAPA flare (vs. controls/bacterial)	6.7 mg/dL	669.2 µg/mL	NR	↑↑SAA vs. controls & bacterial

Footnotes: ^1^Onur reports CRP in mg/dL as printed; probable mislabelling of mg/L (verbatim). Unit heterogeneity: CRP mg/L vs. mg/dL; SAA mg/L vs. µg/mL, not directly comparable. Full between-group comparisons for all groups are included in Supplementary File S5, Supplementary Table 5b.

CRP, C-reactive protein; NR, not reported; PFAPA, periodic fever, aphthous stomatitis, pharyngitis, and adenitis; SAA, serum amyloid A; SURF, syndrome of undifferentiated recurrent fever; mg/L, milligrams per liter; mg/dL, milligrams per deciliter; µg/mL, micrograms per milliliter.

**Table 5:** Acute phase reactants and proteins (cytokines and interferons) (abridged; full version in Supplementary File S5, Supplementary Table 5c).

Study, Year	Contrast	IL-6 (flare/comp.)	IL-1β (flare/comp.)	IL-18 (flare/comp.)	TNF-α (flare/comp.)	IFN-*γ* (flare/comp.)	Key result
Zhu et al., 2026 (34)	PFAPA vs. bacterial	25.1/27.6 pg/mL	NR	NR	2.4/1.9 pg/mL	16.3/3.2 pg/mL	↑↑IFN-γ (*p* < 0.0001); IFN-γ/IL-6 0.7 vs. 0.2 (AUC 0.79); IL-6 NS
Guan et al., 2026 (31)	PFAPA flare (many comparator groups—see: Supplementary File S5)	20.15–114.52 (IQR) pg/mL	NR	NR	2.50–21.82 (IQR) pg/mL	NR	↑IL-6, ↑TNF-α at flare; IL-6 > SURF-periodic (11.8–59.3) > SURF-nonperiodic (5.7–91.1), all NS (full data: Table S5c)
Kapustova et al., 2023 (40)	PFAPA flare vs. interval	6.94/1.52 ng/L	NR	NR	NR	NR	↑IL-6 (*p* < 0.0001)
Onur & Onur, 2023 (39)	—	NR	NR	NR	NR	NR	Cytokines not measured
Lazea et al., 2022 (38)	—	NR	NR	NR	NR	NR	Cytokines not measured
Nakano et al., 2021 (33)	PFAPA vs. recurrent tonsillitis	33.8/46.4 pg/mL	NR	NR	NR	319.0/1.02 IU/mL	↑↑IFN-γ (*p* < 0.01); IL-6 NS
Takeuchi et al., 2019 (35)	PFAPA flare vs. healthy controls	35.0/ < 5 pg/mL	<5/<5 pg/mL	477.1/166.7 pg/mL	0.7/<5 pg/mL	17.8/<5 pg/mL	↑IFN-γ and ↑IL-18 vs. controls; IL-1β undetectable; bacterial comparator IFN-γ <5, IL-6 32.5 pg/mL

Footnotes: Units: IL-6 ng/L (Kapustova) = pg/mL; IFN-γ IU/mL (Nakano) not convertible to pg/mL. Values as published. For Guan, the cell shows PFAPA flare; SURF subgroups summarised in Key result. Full between-group comparisons for all groups: Supplementary File S5, Supplementary Table 5c.

AUC, area under the curve; IFN-γ, interferon gamma; IL-1β, interleukin 1 beta; IL-6, interleukin 6; IL-18, interleukin 18; IQR, interquartile range; IU/mL, international units per milliliter; NR, not reported; NS, not significant; PFAPA, periodic fever, aphthous stomatitis, pharyngitis, and adenitis; pg/mL, picograms per milliliter; SURF, syndrome of undifferentiated recurrent fever; TNF-α, tumor necrosis factor alpha; ng/L, nanograms per liter.

**Table 6:** Additional acute-phase, cellular, and chemokine markers (all included studies):.

Marker	Study	PFAPA value	Comparator value	Direction/significance	Note
Procalcitonin	Lazea et al. (38)	Normal in all patients during flares	Not reported	Normal	Within-cohort observation; no infectious comparator
Procalcitonin	Kapustova et al. (40)	0.12 (0.07–0.55) μg/L (flare)	0.06 (0.04–0.10) μg/L (interval)	↑ flare vs. interval (*p* = 0.0185)	Absolute values remain at/near the normal limit
Ferritin	Zhu et al. (34)	101 (77–148) ng/mL	189 (117–312) ng/mL (bacterial)	↓ in PFAPA (*p* < 0.0001)	Potential exclusion marker for severe infection
Fibrinogen	Zhu et al. (34)	4.5 (3.7–4.9) g/L	5 (4–6.4) g/L (bacterial)	↓ in PFAPA (*p* = 0.0005)	—
Platelets	Zhu et al. (34)	248 (212–278) × 10⁹/L	300 (223.5–378) × 10⁹/L (bacterial)	↓ in PFAPA (*p* = 0.0002)	Independent predictor in composite model
Platelets	Lazea et al. (38)	268,750/mm^3^ (episode)	358,552/mm^3^ (inter-episode)	↓ during flare (*p* = 0.0118)	—
Hemoglobin	Kapustova et al.; Lazea et al.; Onur et al. (38–40)	119 g/L; 11.3 g/dL; 12.07 g/dL	125 g/L; 12.4 g/dL; 12.44 g/dL	Mild ↓ during flare (*p* < 0.0001; *p* = 0.023; *p* = 0.027)	Consistent mild flare-associated anaemia across three cohorts
MPV	Kapustova et al.; Onur et al. (39, 40)	7.7 fL (flare); 8.7 fL	7.5 fL (interval); 9.1 fL (strep)	↑ flare vs. interval (*p* = 0.0186); non-significant vs. strep (*p* = 0.196) 8/7/26 9:46:00 AM	Direction of flare change disputed against earlier reports
NLR	Onur & Onur (39)	2.95 (0.31–14.3)	3.55 (0.26–18.08) (strep pharyngitis)	↓ in PFAPA (*p* = 0.048)	Counterintuitive direction (lower in PFAPA)
Alpha-1 acid glycoprotein	Kapustova et al. (40)	1.51 (1.23–1.65) g/L	0.94 (0.74–1.32) g/L (interval)	↑ flare vs. interval (*p* = 0.0013)	Additional acute-phase reactant
IgD (elevated)	Takeuchi et al.; Kapustova et al. (35, 40)	36.2% elevated; 15.92 mg/L	—; 13.92 mg/L (interval)	↑ in 36.2%; non-significant flare/interval (*p* = 0.36)	Incorporated into the Takeuchi criteria as a supportive item
IL-10	Zhu et al. (34)	2.6 (1.3–4.3) pg/mL	3.5 (2.3–7.2) pg/mL (bacterial)	↓ in PFAPA (*p* = 0.0016)	Component of composite diagnostic model
IL-4	Nakano et al.; Zhu et al. (33, 34)	3.0 pg/mL; 1.5 pg/mL	7.8 pg/mL (tonsillitis); 1.7 pg/mL (bacterial)	↓ in PFAPA (*p* < 0.05) (33); non-significant (34) 8/7/26 9:46:00 AM	Discordant across the two studies
IP-10/CXCL10	Takeuchi et al. (35)	1335.8 ± 7545.5 pg/mL	105.9 (healthy); 335.1 (bacterial)	↑↑ in PFAPA	IFN-γ-induced chemokine; very large SD
MIG (CXCL9)	Takeuchi et al. (35)	1350.8 ± 4881.3 pg/mL	125.0 (healthy); 32.5 (bacterial)	↑↑ in PFAPA	IFN-γ-induced; very large SD
G-CSF	Takeuchi et al. (35)	19.1 ± 13.1 pg/mL	<5 (healthy); 11.2 (bacterial)	↑ in PFAPA > bacterial	—
Neutrophil CD64	Takeuchi et al. (35)	11,421 ± 8,421 molecules	1,385 (healthy); 4,455 (bacterial)	↑↑ in PFAPA	Promising activation marker; requires validation

CD64, cluster of differentiation 64; CXCL10, C-X-C motif chemokine ligand 10; CXCL9, C-X-C motif chemokine ligand 9; G-CSF, granulocyte colony-stimulating factor; IgD, immunoglobulin D; IL-10, interleukin 10; IL-4, interleukin 4; IP-10, interferon gamma-induced protein 10; MIG, monokine induced by gamma interferon; MPV, mean platelet volume; NLR, neutrophil-To-lymphocyte ratio; PFAPA, periodic fever, aphthous stomatitis, pharyngitis, and adenitis; SD, standard deviation; pg/mL, picograms per milliliter; μg/L, micrograms per liter; ng/mL, nanograms per milliliter; g/L, grams per liter; fL, femtoliters.

On QUADAS-2, the three studies reporting diagnostic-accuracy estimates (Onur & Onur, Zhu et al., Takeuchi et al.) were each at high risk of bias in the patient-selection and index-test domains, with the reference-standard and flow-and-timing domains rated high or unclear (Supplementary File S4); their accuracy estimates are therefore interpreted as derivation-stage rather than externally validated.

### 3.1 Characteristics of included studies

Seven original studies published between 2019 and 2026 were included, encompassing 710 pediatric PFAPA patients from 5 countries [China [2 studies], Japan [2 studies], Romania, Slovakia, and Turkey]. The study designs comprised two prospective studies (one clinical and one observational) and five retrospective studies (including cohort, observational, and chart review designs). Diagnostic criteria varied: the Thomas 1999 criteria were used alone or in combination in five studies, the Marshall criteria in three studies, and the Eurofever/PRINTO classification in two studies, with additional guidelines (such as Padeh, Gattorno, or CARRA) supplementing the diagnosis in selected papers. This heterogeneity in diagnostic standards is a limitation affecting between-study comparability.

A summary of the included studies is provided in Table 2.

### 3.2 Biomarker data extraction

Each study in Tables 3–5 occupies a single row; within each marker cell, the value is shown as flare value/comparator value (comparator = asymptomatic interval or comparator disease, as indicated in the Contrast column). These are abridged versions; full between-group comparisons for every group reported in each study are provided in Supplementary File S5 (Supplementary Table 5a–c).

#### 3.2.1 Hematological parameters

Units: White blood cells (WBC), neutrophils (NEUT), and lymphocytes (LYMPH) are reported in the original units; ×10⁹/L and ×10^3^/mm^3^ are numerically equivalent, and/mm^3^ = ×10⁶/L. Takeuchi et al. (35) reported neutrophils only as a percentage (segmented + band), not as an absolute count. “Not reported” denotes a parameter not measured or not provided in the source. Values for Guan et al. (31) are taken from the study's Supplementary Tables 1, 2 (immune indices by subgroup and disease phase).

#### 3.2.2 Inflammatory markers and cytokines

CRP is reported in mg/L in Kapustova et al. (40) and Zhu et al. (34), and in mg/dL in Lazea et al. (38), Nakano et al. (33), Onur & Onur (39), and Takeuchi et al. (35) (1 mg/dL = 10 mg/L); values are presented as published. SAA in mg/L equals μg/mL. IL-6 in pg/mL equals ng/L. IFN-*γ* was reported in IU/mL by Nakano et al. (33), which is not directly convertible to the pg/mL used in Takeuchi et al. (35) and Zhu et al. (34). Calprotectin was measured in serum only, not in stool (Tables 4, 5).

#### 3.2.3 Additional and derived biomarkers reported

Several markers outside the predefined eleven were measured in individual cohorts and are clinically relevant, particularly for differentiating PFAPA from infection. They are summarized here because their omission would understate the available evidence.

### 3.3 Biomarker findings per study

3.3.1 Zhu et al. provided the most rigorous diagnostic-accuracy data in the series, although QUADAS-2 rated the study at high risk of bias in the patient-selection and index-test domains (Supplementary File S4). The IFN-*γ*/IL-6 ratio >0.43 differentiated PFAPA from bacterial infection with an AUC of 0.79, and a composite model combining this ratio with IL-10 and platelet count achieved an AUC of 0.95 (90.3% sensitivity, 89.6% specificity). Both thresholds were derived and tested in the same cohort without external validation. These estimates are best regarded as derivation-stage and require prospective confirmation. IL-6 itself was not statistically different between PFAPA and infection (25.1 vs. 27.6 pg/mL, *p* = 0.722), suggesting that IL-6 has no standalone discriminating value. At the same time, ferritin, fibrinogen, platelets, and ESR were all lower in PFAPA than in bacterial infection (34).

3.3.2 Guan et al. established auto inflammatory disease, and PFAPA in particular, as the leading category of pediatric fever of unknown origin in their cohort, with auto inflammation accounting for 65.8% of all cases. The supplementary Table of Guan et al. provides quantitative immune indices by disease phase. These confirm the characteristic flare signature with hard numbers: in the PFAPA group, the white-cell count rose from 7.13 to 12.35 × 10⁹/L, neutrophils from 3.00 to 8.46 × 10⁹/L, and monocytes from 0.46 to 1.06 × 10⁹/L during attacks. In comparison, lymphocytes fell from 3.38 to 2.61 × 10⁹/L, and interleukin-6 (interquartile range 20.15–114.52 pg/mL) and tumor necrosis factor-α (interquartile range 2.50–21.82 pg/mL) were numerically highest in PFAPA, though all inter-subgroup differences were non-significant.

The same table quantifies the persistent immune dysregulation the authors emphasised: the CD4/CD8 ratio was abnormal (below 1.4 or above 2.0) in 61.2% of PFAPA patients during flares and in a comparable 65.1% during remission, and the absolute CD3, CD4 and CD8 counts were, if anything, lower during flares than in remission, indicating that the lymphocyte disturbance is not confined to the symptomatic episode.

The authors concluded that routine inflammatory markers and peripheral cytokine profiles cannot reliably separate PFAPA from SURF or from infection, a conclusion that the second Guan et al.’s supplementary table (S2) substantiates numerically: across PFAPA and the bacterial, viral and mycoplasma infection groups the acute-phase markers overlapped extensively: PFAPA showed a CRP of 50.5 mg/L (higher than bacterial 26.6 and viral 23.5), an SAA of 238 mg/L (between the viral/bacterial values and the highest value, 304 mg/L, in mycoplasma infection), a white-cell count of 12.35 × 10⁹/L, and an ESR of 48.8 mm/h. The source did not report a formal significance test for every PFAPA-vs.-infection comparison, so non-significance is inferred from overlapping distributions rather than from a powered analysis (31).

3.3.3 Kapustova et al. provided the most complete panel of hematological biomarkers with paired flare and interval sampling. The defining observations were a 53-fold rise in CRP (50 vs. 0.95 mg/L) and a 33-fold rise in SAA (132 vs. 4 mg/L) during flares, a reciprocal leucocyte pattern of neutrophilia and relative lymphopenia at flare giving way to lymphocytosis and eosinophilia in the interval, and a counterintuitive elevation of serum calprotectin during the asymptomatic interval (5.92 vs. 3.64 μg/mL, *p* = 0.036) that the authors interpreted as evidence of ongoing subclinical inflammation.

Procalcitonin rose modestly at flare (0.12 vs. 0.06 μg/L, *p* = 0.0185) but remained at or near the normal limit, so this statistical difference does not undermine the clinical utility of a normal procalcitonin in PFAPA (40).

3.3.4 Onur & Onur systematically evaluated the neutrophil-to-lymphocyte ratio and mean platelet volume. C-reactive protein and ESR were higher in PFAPA than in streptococcal pharyngitis, the neutrophil-to-lymphocyte ratio was paradoxically lower in PFAPA (2.95 vs. 3.55, *p* = 0.048), and age above 49 months was the single best discriminating variable (AUC 0.713) (39). The finding that ESR was higher in PFAPA than in streptococcal pharyngitis is complementary rather than contradictory to Zhu et al., in whom ESR was lower in PFAPA than in a broad bacterial-infection cohort, because streptococcal pharyngitis is a milder, more localized infection than the severe systemic infections that predominate in a general bacterial-infection group (34, 39).

3.3.5 Lazea et al. confirmed the characteristic laboratory signature of neutrophilic leucocytosis, elevated CRP and ESR during episodes and full normalization between them, in agreement with other cohorts.

Its distinctive contribution was that procalcitonin remained normal in every patient during flares, supporting its role in differentiating PFAPA from bacterial infection, and that younger children (onset before one year) experienced more frequent episodes than older children (27 vs. 37.7 days between flares, *p* = 0.0287) (38).

3.3.6 Nakano et al., despite its critically small sample, were the first to propose the IL-4/IFN-*γ* axis as a discriminating signature. Interferon-*γ* was more than three-hundred-fold higher in PFAPA than in recurrent tonsillitis (319 vs. 1.02 IU/mL, *p* < 0.01), while IL-4 was suppressed in PFAPA (3.0 vs. 7.8 pg/mL, *p* < 0.05), and IL-6 and CRP were comparable. The proposed mechanism is that interferon-*γ* suppresses IL-4 transcription, producing a reciprocal pattern; however, the findings are hypothesis-generating only due to the small sample size (33).

3.3.7 Takeuchi et al. provided the most detailed cytokine and neutrophil-activation profiling in the series. Serum amyloid A reached 669 μg/mL during flares and was the most strikingly elevated acute-phase marker. Interferon-*γ* was elevated relative to both healthy controls and bacterial infection (where it was undetectable). IL-1β was below the detection threshold despite the inflammasomal pathophysiology, but IL-18, its more stable caspase-1 co-product, was elevated approximately threefold.

The interferon-induced chemokines IP-10/CXCL10 and MIG/CXCL9 were markedly increased, and neutrophil CD64 expression distinguished PFAPA (11,421 molecules) from both bacterial infection (4,455) and health (1,385). The authors used these data to derive expanded diagnostic criteria with high sensitivity and specificity against both infection and FMF (35).

### 3.4 Cross-study synthesis by biomarker

#### 3.4.1 White blood cell count

Leucocytosis is a consistent feature of PFAPA flares. Kapustova et al. recorded a rise from 8.05 to 11.95 × 10⁹/L between the interval and the flare, Lazea et al. reported a comparable increase from 8,922 to 14,839/mm^3^, and Guan et al.’s supplementary data show an almost identical rise from 7.13 to 12.35 × 10⁹/L, with the change in every case attributable principally to neutrophilia. The marker normalizes between episodes in all three (31, 38, 40). However, the white-cell count has no value in distinguishing PFAPA from infection, since Onur et al. (*p* = 0.231), Zhu et al. (*p* = 0.477), and Nakano et al. all found statistically indistinguishable counts in PFAPA and infectious controls, and Guan et al.’s supplementary comparison concurs, with the PFAPA flare count (12.35 × 10⁹/L) close to that of bacterial (11.88) and mycoplasma (12.08) infection (31, 33, 34, 39). The white-cell count is therefore a well-supported but entirely non-specific indicator of an active flare.

#### 3.4.2 Neutrophils

Neutrophilia tracks the white-cell count closely and rises markedly during flares in all three cohorts with paired data (Kapustova, 3.15 to 6.45 × 10⁹/L; Lazea, 3,386 to 9,977/mm^3^, both *p* ≤ 0.0001; Guan, 3.00 to 8.46 × 10⁹/L), consistent with the known induction of granulopoiesis by interleukin-1, interleukin-6, and granulocyte colony-stimulating factor (23, 31, 35, 38, 40). Monocytosis accompanies neutrophilia in the same three cohorts (e.g., a doubling from 0.46 to 1.06 × 10⁹/L in Guan et al.) (31, 38, 40). Like the total white-cell count, the absolute neutrophil count does not separate PFAPA from infection (34, 39). The more discriminating neutrophil parameter is CD64 expression, which Takeuchi et al. showed to be far higher in PFAPA flares than in bacterial infections or healthy controls; this observation, though promising, derives from a single cohort and requires prospective validation (35).

#### 3.4.3 Lymphocytes

A relative lymphopenia during flares, with a reciprocal lymphocytosis in the interval, is supported by three cohorts. It was statistically significant in Kapustova et al. (2.75 vs. 3.82 × 10⁹/L, *p* = 0.0009) (40). It reproduced as a clear numerical difference in Guan et al. (2.61 during flares vs. 3.38 × 10⁹/L in remission), whereas the corresponding fall in Lazea et al. did not reach significance (*p* = 0.0707) (31, 38). The mechanism proposed in both the PFAPA and the wider literature is interleukin-1-driven redirection of hematopoiesis toward myeloid differentiation at the expense of lymphoid output during acute inflammation (38). However, lymphocyte counts have not been shown to discriminate PFAPA from infection, and the within-flare reduction is modest.

#### 3.4.4 C-reactive protein

CRP is the most widely reported marker of activity. It rises consistently and steeply during flares, with a 53-fold increase in paired data from Kapustova et al. (0.95–50 mg/L), and is completely normalized between episodes in both Kapustova et al. and Lazea et al. (38, 40). The diagnostic question is more nuanced but converges on the theme of non-discrimination. Onur et al. found CRP levels were modestly higher in PFAPA than in streptococcal pharyngitis (*p* = 0.026) (39). Guan et al.’s supplementary data point in the same direction, with CRP during PFAPA flares (50.5 mg/L) numerically higher than in bacterial (26.6 mg/L), viral (23.5 mg/L), and mycoplasma (35.9 mg/L) infections, though without statistical significance (31). Zhu et al., by contrast, found CRP indistinguishable from a broad, more severe bacterial-infection cohort (*p* = 0.474), and Nakano et al. found no difference against tonsillitis (33, 34). The coherent interpretation is that CRP in a PFAPA flare is at least as high as, and often higher than, that of mild-to-moderate infection and equivalent to that of severe bacterial infection, so a raised CRP cannot be used to favor infection over PFAPA. Its discriminating value is negligible and entirely dependent on the severity of the comparator.

#### 3.4.5 Serum amyloid A (SAA)

SAA showed the largest absolute and relative increase of any marker during flares, rising 33-fold in Kapustova et al. (4–132 mg/L) and reaching a mean of 669 μg/mL in Takeuchi et al., making it the most sensitive available marker of PFAPA disease activity (35, 40). Its discriminatory value, however, is consistently limited across two cohorts that were compared with infection (31, 34). Zhu et al. found virtually identical SAA concentrations in PFAPA and bacterial infection (113.2 vs. 116.5 mg/L, *p* = 0.547). Guan et al.'s supplementary data show that PFAPA SAA (238 mg/L) lay above bacterial (86 mg/L) and viral (70 mg/L) infection but below mycoplasma infection (304 mg/L), with no significant autoinflammatory-vs.-infectious difference (31, 34). The highest SAA among the infectious comparators occurred in *Mycoplasma* infection (304 mg/L), although the single highest value overall was in SURF-periodic (321 mg/L); in either case, a very high SAA does not point to PFAPA (31). The marked elevation reported by Takeuchi et al. may reflect cohort-specific severity or assay differences and merits a direct comparative study (35).

#### 3.4.6 Calprotectin

Serum calprotectin was measured in only one cohort, in which it was paradoxically higher during the asymptomatic interval than during the flare (5.92 vs. 3.64 μg/mL, *p* = 0.036). The authors interpreted this as a marker of persistent subclinical inflammation between episodes, a hypothesis that aligns with the demonstration of phase-independent immune dysregulation in Guan et al. and with the persistent tonsillar inflammation described in the wider PFAPA literature (31). As a single-study, counterintuitive finding, it requires independent replication before any conclusion can be drawn, and fecal calprotectin was not assessed in any cohort.

#### 3.4.7 Interleukin-6

Interleukin-6 rose during flares in every cohort that measured it, increasing from 1.52 to 6.94 ng/L in Kapustova et al. (*p* < 0.0001), reaching a mean of 35.0 pg/mL in Takeuchi et al. against undetectable control levels, and spanning an interquartile range of 20.15–114.52 pg/mL in Guan et al. (31, 35, 40). Its diagnostic value, however, is null: Nakano et al. (33.8 vs. 46.4 pg/mL, non-significant), Zhu et al. (25.1 vs. 27.6 pg/mL, *p* = 0.722), and Takeuchi et al. (35.0 vs. 32.5 pg/mL in bacterial infection) all found essentially identical concentrations in PFAPA and infection (33–35). These convergent findings establish interleukin-6 as a sensitive but wholly non-specific marker of acute inflammation that cannot, alone, distinguish autoinflammatory from infectious fever.

#### 3.4.8 Interleukin-1β

Interleukin-1β was reported only by Takeuchi et al., who found it below the assay detection threshold (<5 pg/mL) during flares, in remission, in health, and in bacterial infection alike (35). This represents an apparent paradox, since PFAPA is mechanistically an inflammasome-driven, interleukin-1-mediated disease responsive to interleukin-1 blockade, yet the cytokine is not measurable in peripheral blood. The accepted explanation is that interleukin-1β is short-lived, acts locally in the tonsils and lymph nodes, and is consumed or cleared before venous sampling, often peaking and resolving before the patient reaches the hospital (23, 33, 35). Consequently, serum interleukin-1β does not serve as a routine PFAPA biomarker, and its downstream products better capture the activity of the inflammasome.

#### 3.4.9 Interleukin-18

Interleukin-18, the more stable caspase-1 co-product, was elevated approximately threefold during flares relative to healthy controls in the single cohort in Takeuchi et al. (477 vs. 167 pg/mL). Because it does not require the rapid secretion kinetics of interleukin-1β and persists longer in the circulation, it is a biologically rational surrogate of inflammasome activation in PFAPA (35). The evidence base is a single study with adult controls, and prospective pediatric validation with appropriate comparators is a priority.

#### 3.4.10 Tumor necrosis factor-α

Guan et al. reported an interquartile range of 2.50–21.82 pg/mL during PFAPA flares, with the lower quartile at 2.50 pg/mL, the assay floor, indicating a skewed, low-level elevation present in only a subset of patients (31). This is broadly consistent with the other cohorts that measured it: Takeuchi et al. found concentrations near or below the detection threshold (0.7 ± 2.1 pg/mL), and Zhu et al. found no difference between PFAPA and infection (2.4 vs. 1.9 pg/mL, *p* = 0.323) (34, 35). Serum TNF-α shows at most a modest and inconsistent rise during flares and provides no separation from infection, so it cannot be regarded as a reliable PFAPA biomarker; the low circulating levels are concordant with a disease driven proximally by the inflammasome and the interferon axis rather than by TNF.

#### 3.4.11 Interferon-*γ*

Interferon-*γ* was the most promising discriminating biomarker in this synthesis, although the supporting evidence remains at an early, high-risk-of-bias stage (Supplementary File S4). It was consistently elevated in PFAPA relative to infectious controls across three independent cohorts: more than three-hundred-fold against tonsillitis in Nakano et al. (319 vs. 1.02 IU/mL, *p* < 0.01), measurable in PFAPA but undetectable in bacterial infection in Takeuchi et al. (17.8 vs. <5 pg/mL), and roughly five-fold higher than bacterial infection in Zhu et al. (16.3 vs. 3.2 pg/mL, *p* < 0.0001) (33–35).

The derived IFN-*γ*/IL-6 ratio above 0.43 achieved an AUC of 0.79, and a composite model that included interleukin-10 and platelet count reached an AUC of 0.95 (34). Mechanistically, interferon-*γ* is produced by activated T and natural killer cells as part of a sustained Th1 response in PFAPA, in contrast to the Th2-weighted cytokine pattern of infection, and it simultaneously suppresses interleukin-4 via STAT1 and IRF-2, which explains the reciprocal low interleukin-4 observed by Nakano et al. (33, 41).

The principal caveats are the Nakano cohort's negligible power, the incompatibility of units across studies, and the need for assay standardization. None of the three contributing studies stratified PFAPA patients by IFN-*γ* concentration or reported a corresponding clinical phenotype, and none benchmarked IFN-*γ* or the IFN-*γ*/IL-6 ratio against a monogenic autoinflammatory comparator. The reported cut-off of 0.43 (Zhu et al.) discriminates PFAPA from bacterial infection only, not from any monogenic disease. Elevated serum IFN-*γ* reflects activation of the type II interferon pathway, which is mechanistically and transcriptionally distinct from the type I interferon (IFN-α/*β*) signature that underpins monogenic interferonopathies (34, 42). Whether the marked elevation of IFN-*γ* has any diagnostic overlap with this group cannot be determined from the included studies and remains an open question for future work.

### 3.5 Differentiation of PFAPA from other auto inflammatory diseases

Only two of the seven cohorts reported on the differentiation of PFAPA from other autoinflammatory or undifferentiated recurrent fevers using primary data (31, 35).

Guan et al. compared PFAPA with the syndrome of undifferentiated recurrent fever (SURF) in its periodic and non-periodic forms within a single cohort. During febrile episodes, values overlapped across the three groups: the white-cell count was 12.35, 12.97, and 12.96 × 10⁹/L in PFAPA, SURF periodic, and SURF non-periodic, respectively; the neutrophil count was 8.46, 8.50, and 7.8 × 10⁹/L; and the lymphocyte count was 2.61, 2.88, and 2.69 × 10⁹/L, none of these differences reaching statistical significance. The cytokine interquartile ranges overlapped similarly: interleukin-6, 20.15–114.52, 11.8–59.3, and 5.7–91.1 pg/mL; and tumor necrosis factor-α, 2.50–21.82, 2.50–11.97, and 2.50–18.50 pg/mL, again without significance. The acute-phase reactants were equally indistinguishable, with CRP at 50.5, 41.0, and 46.1 mg/L and SAA at 238, 321, and 228 mg/L in PFAPA, SURF periodic, and SURF non-periodic, respectively. The authors concluded that neither routine laboratory parameters nor peripheral cytokine profiles distinguished PFAPA from SURF. The separation rested on clinical features: fever periodicity, the length of the asymptomatic interval, family history, and the pattern of systemic involvement, rather than on any laboratory marker (between-group values are presented in Tables 3–6 and the Supplementary Material 5) (31).

The only laboratory signal that differed was immunophenotypic: CD19+ B cells were increased in a minority of the SURF periodic group but decreased in 75% of the SURF non-periodic group (lowest absolute CD19 count 241.9 cells/µL in the latter), a pattern separating the two SURF subtypes from one another rather than PFAPA from SURF. In the same cohort, immune dysregulation was not confined to flares: the CD4/CD8 ratio was abnormal in 61.2% of PFAPA patients during flares and in 65.1% during remission, and the absolute counts of CD3, CD4, and CD8 were no higher in remission than in flares (31).

Takeuchi et al. compared PFAPA with typical familial Mediterranean fever (FMF) carrying the M694I mutation in MEFV (*n* = 67). Discrimination was achieved not by any single biomarker but by a multi-item clinical criteria set (the Takeuchi criteria), which separated PFAPA from FMF with 93.8% sensitivity and 95.6% specificity when at least four supportive items were satisfied. For individual markers, serum IgD was elevated in 36.2% of PFAPA patients but not in the M694I FMF cohort (mean 5.4 mg/dL, *n* = 13). The authors noted that IgD cannot distinguish PFAPA from hyperimmunoglobulinaemia D syndrome (mevalonate kinase deficiency), in which it is characteristically elevated. Neutrophil CD64 expression, markedly elevated during PFAPA flares, was reported as not appreciably increased during FMF attacks, although the supporting FMF data were presented only as “data not shown.” The multiplex cytokine panel, including interferon-*γ*, was benchmarked against healthy controls and bacterial infection but not against FMF or any other autoinflammatory disease (35).

The remaining five cohorts did not address this question. Kapustova et al. and Zhu et al. excluded monogenic autoinflammatory diseases by genetic testing but did not retain them as comparator groups (34, 40). Lazea et al. relied solely on clinical criteria, without genetic confirmation (38). Nakano et al. and Onur & Onur compared PFAPA only with infectious entities (33, 39). Kapustova et al. additionally observed that PFAPA lacks any single inflammatory marker correlating with disease activity, in contrast to FMF, in which serum amyloid A fulfils this role (40).

## 4 Discussion

This systematic review yields three principal findings. Circulating markers of inflammation rise reproducibly during PFAPA flares but, with a single exception, do not distinguish flares from infections. Interferon-*γ*, and particularly the IFN-*γ*/IL-6 ratio, is the only signal with consistent discriminating performance, although the supporting evidence is at high risk of bias. No biomarker has yet separated PFAPA from other autoinflammatory diseases, against which differentiation still depends on clinical phenotype and genetic testing.

These observations fit a coherent immunological model. PFAPA behaves as a disorder of innate immunity in which inflammasome activation, although central to pathogenesis, is poorly reflected by peripheral interleukin-1β, so the more durable caspase-1 product interleukin-18 is a better, though so far single-cohort, surrogate of inflammasome activity (23, 35). Downstream of the inflammasome, the dominant circulating signal is a Th1/interferon axis, evidenced by elevated interferon-*γ* and its induced chemokines IP-10/CXCL10 and MIG/CXCL9. This axis, rather than the generic acute-phase cytokines, most reliably separates PFAPA from infection (33–35).

By contrast, interleukin-6 and tumor necrosis factor-α are either non-discriminating (interleukin-6) or only modestly and inconsistently raised (TNF-α, with a lower quartile at the assay floor even during flares) (31). The acute-phase response, mediated by interleukin-6 and reflected in CRP and SAA, is intense during flares but is also shared with infection and therefore non-discriminatory (33–35, 40). The disturbance is not confined to the symptomatic flare. The phase-independent CD4/CD8 abnormality and the paradoxical inter-episode elevation of serum calprotectin (Section 3.5) both indicate persistent, subclinical dysregulation, consistent with the persistent tonsillar inflammation described in the broader literature (27, 31, 40).

A clear conceptual separation between biomarkers of disease activity and biomarkers of differential diagnosis emerges, a distinction not always maintained in the primary studies. As detailed in Sections 3.4.1–3.4.7, CRP, SAA, and the white-cell and neutrophil counts are well supported as activity markers but, being shared with infection, do not withstand the more pressing test of distinguishing a flare from acute infection (33–35, 38–40). This is the central diagnostic problem of PFAPA, and it explains both the persistent diagnostic delay, a median of 12 months in Zhu et al. and over 2 years in Lazea et al., and the repeated, inappropriate antibiotic exposure documented across cohorts (34, 38, 39). Interferon-*γ* was the only biomarker with consistent discriminating performance, elevated in PFAPA relative to infection across three independent cohorts spanning two countries and a decade (33–35). Its transformation into the IFN-*γ*/IL-6 ratio by Zhu et al. and incorporation into a composite model with interleukin-10 and platelet count (AUC 0.95) is the finding with the clearest potential clinical application, aligning with the IP-10/CXCL10 data of Takeuchi et al. and the IL-4/IFN-*γ* reciprocity of Nakano et al. (33–35). Its biological plausibility is strong, a sustained Th1/interferon response being mechanistically distinct from the profile of pyogenic infection. However, all three diagnostic-accuracy studies were rated at high risk of bias on QUADAS-2 in the patient-selection and index-test domain, reflecting two-gate sampling and in-sample-derived thresholds. The reported accuracy estimates, including the AUC of 0.95, should be interpreted as derivation-stage rather than externally validated [Supplementary File S4 (37),].

Several findings require cautious interpretation. The serum calprotectin paradox, higher between flares than during them, is intriguing and consistent with the phase-independent dysregulation noted above, but rests on a single cohort and a single, less well-validated assay and cannot yet be regarded as established (31, 40). The interleukin-1β paradox, in which the cytokine is undetectable despite an interleukin-1-responsive disease, is biologically explicable but forces indirect probing of the inflammasome (23, 35). The three-fold elevation of interleukin-18 in Takeuchi et al. is the most promising indirect marker, but remains unreplicated and was benchmarked against adult rather than pediatric controls; the neutrophil CD64 signal is similarly promising and similarly unvalidated (35).

The apparent disagreement over the erythrocyte sedimentation rate, higher in PFAPA than in streptococcal pharyngitis in Onur et al. but lower than in a broad bacterial cohort in Zhu et al., reflects comparator severity rather than a true inconsistency. Streptococcal pharyngitis elicits a weaker systemic response than the severe infections dominating a general bacterial group (34, 39). Guan et al. illustrate the same principle directly, showing that within one cohort, PFAPA had the highest CRP, mycoplasma had the highest SAA, and the sedimentation rate and white-cell count were essentially equal across groups (Section 3.5) (31). The diagnostic performance of any PFAPA biomarker, therefore, depends on the chosen comparator, so cross-study comparisons of discriminating metrics are unreliable when comparator groups differ in composition and severity. A high acute-phase response, including markedly elevated CRP or SAA, should never be used to favor infection over a PFAPA flare.

A further, and arguably more fundamental, gap concerns the differentiation of PFAPA from other autoinflammatory diseases. This comparison that matters most once infection has been excluded, yet one that the literature scarcely addresses. Where it was made directly, biomarkers failed. Against SURF, routine laboratory parameters and peripheral cytokine profiles overlapped completely, leaving fever periodicity and family history to carry the entire diagnostic burden (Section 3.5) (31). Against FMF, discrimination was achieved by a clinical criterion set rather than any single analyte, and the biomarker-specific evidence was weak or unverifiable. Raised IgD favoring PFAPA only weakly and unable to separate it from mevalonate kinase deficiency, and a CD64 signal whose FMF comparison was reported only as “data not shown” (Section 3.5) (35). That no cytokine, including the otherwise promising interferon-*γ*, has been benchmarked against a monogenic comparator is a striking omission, because it is precisely these genetically defined entities from which PFAPA most needs to be distinguished in specialist practice.

The interferon-*γ* signal that separates PFAPA from infection may or may not survive comparison with FMF, HIDS, or TRAPS, in which IL-1-driven and interferon pathways are also active (43). A related question is whether children with markedly elevated IFN-*γ* could instead represent a monogenic interferonopathy. The classic interferonopathies (e.g., CANDLE/PRAAS, SAVI, Aicardi–Goutières syndrome) are defined by dysregulated type I interferon (IFN-α/*β*) signaling and an interferon-stimulated gene expression signature, which is mechanistically distinct from the type II interferon (IFN-*γ*) elevation observed in the included PFAPA studies (42). None of the included studies tested for a type I interferon signature or reported the clinical features of their highest-IFN-*γ* patients, so any overlap between PFAPA's IFN-*γ* elevation and the interferonopathies remains a biologically motivated but currently untested hypothesis rather than a conclusion this review can draw. On present evidence, the differentiation of PFAPA from other autoinflammatory diseases remains a clinical and genetic exercise rather than a biomarker-based one. It is the principal unresolved question in the field.

The limitations of the underlying literature are substantial and constrain every conclusion. A formal meta-analysis was not feasible due to heterogeneity across comparator groups, assay platforms, units, and reporting formats. Diagnostic criteria were inconsistent across the analyzed articles: five studies applied the Thomas 1999 criteria alone or in combination, three applied Marshall's original criteria, two applied the Eurofever/PRINTO classification, and Takeuchi et al. additionally validated a new criteria set within the same cohort used to derive it, a form of criterion contamination (Table 2) (35). Timing of blood sampling relative to fever onset also varied and was frequently a source of confounding rather than a fixed protocol element: in Zhu et al., the median sampling day was 2 in PFAPA vs. 6 in bacterial infection, leaving residual confounding despite a sensitivity analysis restricted to a 24–72-hour window (34). In Kapustova et al., inter-flare samples were drawn at least two weeks after the attack (40). In Nakano et al., PFAPA samples were drawn 1–4 days after onset, whereas in recurrent tonsillitis, they were drawn 2–3 days after onset (33). Laboratory assay platforms for the same analyte also differed across cohorts: cytokines were measured by cytometric bead array in Takeuchi et al. (35), a different multiplex bead platform (Flowcytomix) in Zhu et al. (34), conventional ELISA for interleukin-6 and TNF-α in Guan et al. (31), and an unspecified reference-laboratory immunoassay in Nakano et al. (33). Interferon-*γ* was reported in IU/mL in one study and pg/mL in two others, preventing direct pooling (33–35).

Several study-specific data-quality issues further limit interpretation. Cytokines were measured in only five of the seven cohorts, and the study with the most striking cytokine findings, Nakano et al., enrolled only three PFAPA patients, rendering its results hypothesis-generating at best (31, 33–35, 40). Healthy comparators, when present (Takeuchi et al.), were adults rather than age-matched children (35). The inter-episode WBC range in Lazea et al. is internally implausible (38). The CRP units in Onur et al. appear mislabelled (39). A limitation specific to Guan et al. is that supplementary cytokine concentrations are given only as interquartile ranges, without medians, so the central tendency of interleukin-6 and TNF-α cannot be recovered (31). Takeuchi et al.'s cytokine means are accompanied by standard deviations exceeding the means, indicating skewed distributions for which median reporting would have been preferable (35).

At the review level, five of the seven studies were retrospective, and most were single-center referral cohorts, which carry the attendant risks of selection and information bias. The search was limited to two databases (PubMed and Scopus) and to English-language reports, for the reasons given in Section 2.4. It did not include Embase, Web of Science, the Cochrane Library, trial registries, grey literature, or citation searching, so studies indexed elsewhere or appearing after the 07 June 2026 cut-off may have been missed. These findings, therefore, represent a current rather than exhaustive synthesis.

In current practice, the evidence supports using CRP and SAA to confirm and monitor PFAPA flares while explicitly cautioning against their use to separate PFAPA from infection (33–35, 40). A normal procalcitonin during a febrile episode, as observed by Lazea et al. and supported by the near-normal values of Kapustova et al., may favor PFAPA over bacterial infection and is a low-cost, widely available adjunct (38, 40). Among emerging markers, the IFN-*γ*/IL-6 ratio is the most clinically promising and, in the composite model of Zhu et al., approaches the performance required for diagnostic use. Although it was derived and tested within a single cohort at high risk of bias (Supplementary File S4), it cannot be recommended for routine use until prospectively and externally validated (34).

Translating an interferon-*γ*-based approach into everyday practice also requires considering test accessibility. None of the included studies reported cost, turnaround time, or point-of-care availability, so the following is offered as a general laboratory-medicine context rather than a finding of this review. Unlike CRP and procalcitonin, which run on automated chemistry platforms with same-hour results in essentially any hospital laboratory, IFN-*γ* in the contributing cohorts was measured by send-out or batch-run multiplex and reference-laboratory techniques (see the heterogeneity discussion above), methods typically available only at tertiary or reference centres, with turnaround measured in days and higher per-test cost. This accessibility gap is a barrier to clinical adoption, independent of the validation gap discussed above.

The research priorities that follow are prospective, multicentre studies of interferon-*γ* and the IFN-*γ*/IL-6 ratio with harmonized assays and units, ideally on a common multiplex platform across sites, using a pre-specified rather than in-sample-derived cut-off and a formal external validation cohort, temporally or geographically independent from the derivation sample. Such studies should employ a standardized, prospectively defined biomarker panel and sampling protocol, a fixed post-onset sampling window for both flare and interval samples, and, at a minimum, CRP, SAA, procalcitonin, IFN-*γ*, IL-6, and IL-18 measured in every participant, so that future cohorts can be pooled or compared directly. Independent replication of the serum calprotectin and neutrophil CD64 findings, measurement of interleukin-18 in adequately powered pediatric cohorts with age-matched comparators, and longitudinal characterization of the inter-episode state are also needed (31, 35, 40).

The highest priority, however, is head-to-head biomarker comparison of PFAPA against monogenic autoinflammatory diseases such as FMF, mevalonate kinase deficiency (MKD), and TRAPS, because no cytokine has yet been tested against these entities. It is this differentiation, rather than the distinction from infection, that most often determines specialist management (31, 35).

## 5 Conclusions

This synthesis of seven cohorts comprising 710 children with PFAPA demonstrates that the acute-phase reactants CRP and SAA are robust markers of disease activity but cannot distinguish PFAPA flares from acute infections, the principal unmet diagnostic need in this condition. Interferon-*γ*, and especially the IFN-*γ*/IL-6 ratio, is the most promising discriminating biomarker identified, supported by consistent direction across three independent cohorts. However, the supporting accuracy estimates derive from single cohorts at high risk of bias (two-gate designs with in-sample-derived thresholds) and require prospective, externally validated confirmation before clinical use.

Because the entire evidence base for this review comprises only seven studies and 710 children, with most individual biomarkers supported by one to three cohorts, the discriminatory conclusions for interferon-*γ*, interleukin-18, neutrophil CD64, serum calprotectin, and the composite biomarker models alike should all be regarded as preliminary and hypothesis-generating rather than practice-changing. By GRADE, the certainty of the interferon-*γ* discrimination evidence is low, and it is low or very low for every other biomarker's discrimination evidence (Supplementary File S3). Only CRP and SAA, as activity markers, reach moderate-to-high certainty. This gap between promising point estimates and low underlying certainty reflects small sample sizes, methodological heterogeneity, and incompatible assay units.

Interleukin-1β is uninformative in serum despite the inflammasomal basis of the disease, and interleukin-18, neutrophil CD64, and a normal procalcitonin are biologically rational but inadequately validated alternatives.

No circulating biomarker has been shown to distinguish PFAPA from other autoinflammatory diseases: the single direct comparison with SURF found that routine and cytokine markers fail to do so. The differentiation depends on clinical phenotype, and discrimination from familial Mediterranean fever was achieved by a clinical criterion set rather than any analyte, leaving the differentiation most relevant to specialist practice without a biomarker-based solution.

Until prospective, multicentre studies confirm their diagnostic performance, no single circulating biomarker can be recommended for routine PFAPA diagnosis.

At present, PFAPA remains a clinical diagnosis, supported by the characteristic pattern of elevated acute-phase reactants that normalize between episodes. Exclusion of monogenic auto inflammatory diseases, ideally via genetic testing, remains essential.

## Data Availability

The original contributions presented in the study are included in the article/supplementary material, further inquiries can be directed to the corresponding author.

## Glossary

AUC: area under the curve
CARRA: childhood arthritis and rheumatology research alliance
CD3/CD4/CD8/CD19/CD64: cluster of differentiation 3/4/8/19/64
CRP: C-reactive protein
CXCL9: C-X-C motif chemokine ligand 9 (MIG)
CXCL10: C-X-C motif chemokine ligand 10 (IP-10)
DOI: digital object identifier
ESR: erythrocyte sedimentation rate
FMF: familial mediterranean fever
FUO: fever of unknown origin
G-CSF: granulocyte colony-stimulating factor
GRADE: grading of recommendations, assessment, development and evaluations
HIDS: hyperimmunoglobulinaemia D syndrome
IFN-*γ*: interferon gamma
IgD: immunoglobulin D
IL-1, IL-1β, IL-4, IL-6, IL-10, IL-18: interleukin-1, −1*β*, −4, −6, −10, −18
IP-10: interferon-*γ*-induced protein 10 (CXCL10)
IQR: interquartile range
IRF-2: interferon regulatory factor 2
IU: international unit
JBI: Joanna briggs institute
MEDLINE: medical literature analysis and retrieval system online
MEFV: mediterranean fever (Gene)
MIG: monokine induced by interferon-*γ* (CXCL9)
MKD: mevalonate kinase deficiency
MPV: mean platelet volume
NLR: neutrophil-to-lymphocyte ratio
NR: not reported
NS: not significant
PECO: population, exposure, comparator, outcome
PFAPA: periodic fever, aphthous stomatitis, pharyngitis, and cervical adenitis
PMID: pubmed identifier
PRINTO: paediatric rheumatology international trials organisation
PRISMA: preferred reporting items for systematic reviews and meta-analyses
PROSPERO: international prospective register of systematic reviews
QUADAS-2: quality assessment of diagnostic accuracy studies, version 2
ROC: receiver operating characteristic
SAA: serum amyloid A
SD: standard deviation
STAT1: signal transducer and activator of transcription 1
SURF: syndrome of undifferentiated recurrent fever
Th1: T-helper type 1 (cell)
TNF-α: tumor necrosis factor alpha
TRAPS: tumor necrosis factor receptor-associated periodic syndrome
WBC: hite blood cell (Count).
