# Supplementary material for: Inflammatory biomarkers in pediatric PFAPA syndrome: a systematic review
Source: Front Pediatr. 2026 Sep 3;14:1934055. doi: 10.3389/fped.2026.1934055 (PMC13582404; doi:10.3389/fped.2026.1934055)
Supplement: Supplementary file 1 [file Supplementaryfile1.docx]

**Supplementary File S1. Systematic Review Protocol**

**Title:** Inflammatory Biomarkers in Pediatric PFAPA Syndrome: A Systematic Review.

*Authors: Paweł Treichel^1,2^; Sylwia Kołtan^1^; Jan Styczyński^1^*

1. *Department of Pediatrics, Hematology, Oncology, Immunology, and Transplantology, Nicolaus Copernicus University in Toruń, Collegium Medicum in Bydgoszcz*
2. *Doctoral School of Medical and Health Sciences, Nicolaus Copernicus University in Toruń, Collegium Medicum in Bydgoszcz*

**1. Background**

Periodic fever, aphthous stomatitis, pharyngitis, and cervical adenitis (PFAPA) syndrome is the most common autoinflammatory periodic fever of childhood.
Yet, it remains a diagnosis of exclusion because no validated confirmatory biomarker exists. Two clinical problems dominate management. The first is the monitoring
of disease activity, and distinguishing the febrile attack from the asymptomatic interval. The second, and more demanding, is the differentiation of a PFAPA flare from acute infection, which the syndrome shares its clinical and routine-laboratory phenotype with, leading to diagnostic delay and repeated, unnecessary antibiotic exposure.
A separate and largely unaddressed question is whether any biomarker
can distinguish PFAPA from other autoinflammatory diseases. A range of circulating markers — hematological indices, acute-phase reactants, and cytokines — has been proposed, but their performance has not been systematically synthesized. This review summarizes the current evidence on circulating biomarkers in pediatric PFAPA, appraises the methodological quality of the primary studies, and grades the certainty of the resulting evidence.

**2. Objectives and PECO Question**

Because this is a biomarker (not an intervention) review, the question is framed using the PECO structure.

| **PECO Component** | **Specification** |
| --- | --- |
| **Population (P)** (Who?) | Pediatric patients aged 0–18 years with confirmed PFAPA syndrome diagnosed by validated clinical criteria (Thomas 1999, Eurofever/PRINTO 2019, Takeuchi 2019, or equivalent), with no prior diagnosis of monogenic autoinflammatory disease (FMF, HIDS, TRAPS, CAPS, cyclic neutropenia), primary immunodeficiency, malignancy, or systemic autoimmune disorder.  **Excluded:** Adults (>18 years); studies in which PFAPA patients could not be analysed separately from other diagnoses.  *Note: SURF and monogenic autoinflammatory diseases (e.g. FMF) were not an exclusion at the patient level. They are retained as comparator groups for the differential-diagnosis question (see Comparison).* |
| **Exposure / Index (E)** (What biomarkers?) | Quantitative measurement of serum or blood inflammatory biomarkers during febrile episodes and/or asymptomatic inter-flare periods:  **Hematological:** WBC, neutrophils, lymphocytes, monocytes, eosinophils, NLR, MPV, platelets, haemoglobin, CD64 expression on neutrophils/monocytes.  **Acute-phase proteins:** CRP, SAA, serum calprotectin.  **Cytokines/chemokines:** IL-1, IL-6, IL-18, TNF-α, IFN-γ and derived ratios (IFN-γ/IL-6). |
| **Comparison (C)** (Compared to what?) | Disease phase (febrile attack versus asymptomatic interval) and/or an external comparator group (acute infection, SURF, or another autoinflammatory disease such as familial Mediterranean fever). |
| **Outcomes (O)** (What results?) | I. The change in biomarker concentration during the flare versus the interval (disease-activity monitoring). II. Discrimination of PFAPA from infection. III. Discrimination of PFAPA from other autoinflammatory diseases; and, where reported, diagnostic-accuracy metrics (sensitivity, specificity, area under the curve). |

**PECO-based Research Question:**

In pediatric patients (0–18 years) with confirmed PFAPA syndrome, which inflammatory biomarkers are significantly elevated during febrile episodes compared to asymptomatic inter-flare periods or to children with confirmed bacterial infection, and which individual or combined biomarker profiles optimally discriminate PFAPA from acute infectious fever?

**3. Methods**

**3.1. Design**

This systematic review was conducted in accordance with the PRISMA 2020 statement. Owing to substantial heterogeneity in comparator groups, assay platforms, units of measurement, and reporting formats, a structured qualitative (narrative) synthesis was undertaken, and no meta-analysis was prespecified. The certainty
of evidence was rated using the GRADE approach. The PRISMA 2020 flow diagram
is presented in the main text (Figure 1), and a completed PRISMA checklist accompanies the review (Supplementary Table S1).

**3.2 Eligibility Criteria**

**a) Inclusion criteria:**

- Original clinical studies (prospective or retrospective cohorts, cross-sectional studies, case-control designs, or randomized controlled trials)
- Pediatric population (age 0–18 years) with confirmed PFAPA syndrome according to any validated diagnostic criteria (Thomas 1999, Vanoni/Gattorno 2018, Eurofever/PRINTO 2019, Takeuchi 2019 or equivalent)
- Reporting quantitative or semi-quantitative data for at least
  one of the following biomarkers: WBC, neutrophils, lymphocytes, CRP, SAA, serum calprotectin, IL-6, IL-1β, IL-18, TNF-α, or IFN-γ
- Published in English between 1 January 2018 and 7 June 2026
- Full text available

**b) Exclusion criteria:**

- Review articles, meta-analyses, editorials, commentaries, and letters
- Case reports and case series with fewer than 5 PFAPA patients
- Studies with exclusively adult populations (age >18 years)
- Animal studies
- Studies in which PFAPA served only as a reference/comparison group,
  with no PFAPA-specific biomarker values reported.
- Conference abstracts without corresponding full-text publication
- Studies without extractable biomarker data for PFAPA patients
  3.3 **Information Sources and Search Strategy**
- Bibliographic databases: PubMed/MEDLINE (National Library of Medicine, USA) and Scopus (three complementary query blocks in each database). These two databases were selected a priori because they jointly provide broad coverage of the pediatric rheumatology/immunology literature relevant to this review. This restriction is acknowledged as a limitation in the main text (Discussion, Section 4).
- Data range: 1 January 2018 – 07 June 2026.
- Filters: No automated database filters were applied during the search process.
- Searches were performed using English-language terms.
- No additional searches (reference-lists checking, citation tracking in Google Scholar or Web of Science, or grey literature sources) were conducted.
  3.4 **Study Selection**
- The deduplication process, as well as the subsequent screening and analysis, was conducted manually and automatically using the Rayyan platform
  (web-based software for systematic review screening).
- Two independent reviewers screened and analyzed titles and abstracts against eligibility criteria.
- Full texts were retrieved and assessed for all records that passed the initial screening.
- Disagreements were resolved by consensus or by consultation with a third reviewer.
- The selection process is documented in the PRISMA 2020 flow diagram
  (Figure 1).
  3.5 **Data Extraction**
- A standardized extraction form was applied to each study, and two reviewers independently extracted the data.
- Extracted items: first author and year; DOI and, where available, PubMed identifier; country; study design; number of patients with PFAPA; diagnostic criteria; mean or median age; disease phase assessed and comparator group; and numerical values (mean ± SD or median [IQR], as originally published)
  for each predefined biomarker.
- Where values were unavailable from the abstract, the full text and, where relevant, the supplementary files were accessed (as for the immune indices
  of Guan et al. in Supplementary Table S1).
- When a value remained unavailable despite full-text and supplementary access, the entry was coded as "Not reported" (NR). Discrepancies were resolved by discussion.
  3.6 **Risk of Bias Assessment**
- Methodological quality was appraised with the Joanna Briggs Institute (JBI) Critical Appraisal Checklist for Analytical Cross-Sectional Studies, applied uniformly to all included cohorts.
- For the two studies that used paired within-patient flare-versus-interval sampling, the equivalent JBI cohort items were also considered when interpreting the confounding domains.
- In addition to the JBI appraisal, the three studies that reported diagnostic-accuracy estimates (Onur & Onur, Zhu et al., Takeuchi et al.) were appraised with QUADAS-2 across its four domains. QUADAS-2 was not extended to the remaining studies, which reported only within-group or descriptive comparisons. The QUADAS-2 appraisal is reported in Supplementary File S4.
- Each study was assessed by two reviewers (P.T., S.K.), with disagreements resolved by consensus or a third reviewer (J.S.). The item-level appraisal
  and overall judgments are reported in the main text and summarised
  in Supplementary File S2 and Supplementary Table S3.
  3.7 **Data Synthesis**
- The primary synthesis was narrative, owing to heterogeneity in study designs, comparator groups, assay platforms, units, and reporting formats.
- Each study was treated as an independent unit, and no values were pooled; where feasible, results are summarised per biomarker and per question
  as medians, interquartile ranges, and ranges.
- No formal meta-analysis was prespecified.
- The certainty of evidence was rated with GRADE for the disease-activity
  and infection-discrimination questions; the differentiation of PFAPA from other autoinflammatory diseases was assessed narratively.

**4. Planned Results Reporting**

- General description of the included studies.
- Biomarker data by disease phase and comparator.
- Cross-study synthesis per biomarker and an immunological interpretation.
- Differentiation of PFAPA from infection and from other autoinflammatory diseases.
- Risk-of-bias appraisal (JBI for all studies; QUADAS-2 for the diagnostic-accuracy studies) and overall judgments.
- GRADE evidence profile.
- Discussion of strengths, limitations, and priorities for future research.

**5. Bias Minimization Measures**

- A priori definition of the PECO, eligibility criteria, predefined biomarker panel, and analysis plan.
- Independent duplicate screening and data extraction.
- Resolution of disagreements by consensus or third-party adjudication.
- Standardized data-collection forms.
- Risk-of-bias assessment with validated tools (JBI for all studies; QUADAS-2
  for the diagnostic-accuracy studies) and certainty rating with GRADE.

**6. Deviations from Protocol**

- No major deviations were introduced during the review.
- The supplementary table of immune indices for Guan et al. was obtained during the review, and its values were incorporated into the synthesis,
  risk-of-bias appraisal, and GRADE profile.
- The lack of prospective registration in PROSPERO or a similar repository
  is acknowledged as a limitation.

**7. Dissemination**

The results of this systematic review are presented in the main manuscript.
