# Supplementary material for: Inflammatory biomarkers in pediatric PFAPA syndrome: a systematic review
Source: Front Pediatr. 2026 Sep 3;14:1934055. doi: 10.3389/fped.2026.1934055 (PMC13582404; doi:10.3389/fped.2026.1934055)
Supplement: Supplementary file 2 [file Supplementaryfile2.docx]

**Supplementary File S2. Risk of Bias Assessment (Joanna Briggs Institute Critical Appraisal Tool)**

1. Instrument and rationale:

Methodological quality was appraised with the Joanna Briggs Institute (JBI) Critical Appraisal Checklist for Analytical Cross-Sectional Studies, applied uniformly to all seven cohorts to permit direct cross-study comparison. This instrument was selected because the central analysis in every included study is a group-wise comparison of circulating biomarker concentrations measured at a defined time point, whether PFAPA versus an infectious or autoinflammatory comparator, or the flare versus the asymptomatic interval, which corresponds most closely
to an analytical cross-sectional design.

For the two studies (Kapustova et al. and Lazea et al. [1,2]) that used paired within-patient flare-versus-interval sampling, a cohort logic also applied, and the equivalent JBI cohort items were considered when interpreting the confounding domains; the cross-sectional checklist was nonetheless retained throughout for comparability. In the biomarker context, the JBI terms exposure (items 3–4) and outcome (item 7) were operationalized as assignment to the diagnostic group according to validated PFAPA criteria and measurement of circulating biomarkers, respectively. Each item was rated as "Yes", "No", "Unclear", or "Not applicable". A full checklist with critical appraisal is available in Table 1. JBI risk of bias summary is presented in a graphic form in Figure 1.

Because the JBI cross-sectional checklist does not capture the domains specific
to diagnostic test-accuracy research, the three studies that reported diagnostic-accuracy estimates (Onur & Onur [3], Zhu et al. [4], Takeuchi et al. [5]) were also appraised with QUADAS-2 [6] (Supplementary File S4). The comparatively favorable JBI ratings of these studies therefore coexist with a high QUADAS-2 risk of bias, because the two instruments interrogate different features:
JBI the analytical-cross-sectional conduct, QUADAS-2 the patient-selection, index-test,
reference-standard, and flow-and-timing domains that govern diagnostic accuracy.

In line with JBI guidance, no arbitrary numeric threshold was used to dichotomize quality; the number of satisfied items was reported descriptively, and an overall risk of bias
was assigned to each study based on the number and seriousness of the domains of concern.

2. The eight appraisal items:

1. Were the criteria for inclusion in the sample clearly defined?
2. Were the study subjects and the setting described in detail?
3. Was the exposure measured validly and reliably?
4. Were objective, standard criteria used for measurement of the condition?
5. Were confounding factors identified?
6. Were strategies to deal with confounding factors stated?
7. Were the outcomes measured validly and reliably?
8. Was an appropriate statistical analysis used?

3. Item-level appraisal:

Table 1. JBI critical appraisal of the seven included studies (analytical cross-sectional checklist)

| Study | Q1 | Q2 | Q3 | Q4 | Q5 | Q6 | Q7 | Q8 | Items met | Overall risk of bias |
| --- | --- | --- | --- | --- | --- | --- | --- | --- | --- | --- |
| Guan et al., 2026 [7] | Y | Y | Y | Y | U | N | Y | Y | 6/8 | Moderate |
| Zhu et al., 2026 [4] | Y | Y | Y | Y | Y | Y | Y | Y | 8/8 | Low to moderate |
| Kapustova et al., 2023 [1] | Y | Y | Y | Y | Y | Y | Y | Y | 8/8 | Low |
| Onur & Onur, 2023 [3] | Y | Y | Y | Y | Y | N | U | Y | 6/8 | Moderate |
| Lazea et al., 2022 [2] | Y | Y | U | Y | U | Y | U | Y | 5/8 | Moderate to high |
| Nakano et al., 2021 [8] | Y | U | U | Y | N | N | U | U | 2/8 | High |
| Takeuchi et al., 2019 [5] | Y | Y | Y | Y | Y | U | Y | U | 6/8 | Low to moderate |

*Y = Yes (criterion met, low concern); N = No (criterion not met, high concern); U = Unclear. Q1–Q8 correspond to the eight items listed in Section 2.*

4. Study-level appraisal

a) Guan et al., 2026 [7]. This cohort defined clear inclusion criteria through the Modified Marshall criteria and the Eurofever/PRINTO framework. It provided a detailed tertiary-center description (items 1, 2), with a valid, large-scale diagnostic assignment (items 3, 4). The appraisal is weakened by confounding acknowledged for the referral pattern but not controlled in the biomarker comparison, where the infectious and SURF comparator groups were neither age-matched
nor adjusted for sampling time (item 5 unclear; item 6 not met). The Supplementary Tables S1
and S2 of Guan et al. article provide, biomarker measurement can be appraised and is judged valid and reliable: the haematological parameters and acute-phase reactants were obtained by standard methods and the lymphocyte subsets by flow cytometry, with full mean and standard-deviation reporting by disease phase and by comparator group (item 7); the only residual measurement concern is that interleukin-6 and TNF-α are reported as interquartile ranges without medians.
The statistical methods were appropriate (item 8). With six of the eight items satisfied, the study was judged to be at moderate risk of bias, the residual concern being the absence
of any age-matching or statistical adjustment for confounding in the PFAPA-versus-infection
and PFAPA-versus-SURF comparisons.

b) Zhu et al., 2026 [4]. This study met all eight items: it applied rigorous Eurofever/PRINTO
and CARRA inclusion criteria with genetic exclusion of monogenic disease (items 1, 3, 4), described its subjects and setting in detail (item 2), explicitly identified the sampling-time confounder (item 5), and combined multivariable logistic regression with a sensitivity analysis restricted to a 24–72-hour sampling window and formal calibration testing (items 6, 8), applied
to validly measured cytokines (item 7). Because the JBI cross-sectional checklist lacks a domain
to capture the retrospective single-center design or the residual confounding by sampling time that persists despite the sensitivity analysis, a half-step of caution was added beyond the item tally, and the study was judged to be at low-to-moderate risk of bias.

c) Kapustova et al., 2023 [1]. This prospective study satisfied all eight items. The inclusion criteria, setting, and subjects were clearly described (items 1, 2), and the within-patient paired flare-versus-interval design provided a valid and reliable exposure assignment (items 3, 4) that inherently controls between-subject confounding (items 5, 6), reinforced by the genetic reclassification
of two familial Mediterranean fever cases. Biomarkers were measured with standard assays
(item 7) and analyzed appropriately (item 8). The only residual concerns are the less-validated serum calprotectin assay and the unbalanced allocation to ketotifen. This could influence interval measurements, which are minor and do not alter the judgment. This was the strongest study
in the series and was judged to be at low risk of bias.

d) Onur & Onur, 2023 [3]. This prospective study clearly defined its inclusion criteria, including
a negative rapid streptococcal antigen test, and described its setting and subjects in detail
(items 1 and 2), and provided a valid case definition (items 3 and 4). Confounding was identified, since the age difference between the groups was reported (item 5). Still, it was not controlled
in the biomarker comparisons despite age being the strongest single discriminating variable
(area under the curve 0.713), so the strategy item is not met (item 6). Biomarker-measurement validity is uncertain because C-reactive protein is labeled in mg/dL, whereas the magnitude
of the values implies mg/L, an unacknowledged probable unit error (item 7 unclear). The statistical analysis, including receiver operating characteristic analysis and a documented power calculation, was appropriate (item 8). The study was judged to be at moderate risk of bias.

e) Lazea et al., 2022 [2]. The inclusion criteria and the single-center setting were clearly described (items 1 and 2), and the paired episode-versus-inter-episode structure controls for within-subject confounding (item 6). The diagnostic assignment is rated unclear (item 3) because monogenic periodic fevers were not excluded by genetic testing, a limitation the authors acknowledge,
and confounding was only partly addressed (item 5 unclear). Biomarker-measurement validity
is uncertain (item 7): the reported inter-episode white-cell range of 5,000–138,000/mm³
is physiologically implausible and almost certainly a transcription error, and cytokines and serum amyloid A were not measured. The statistical analysis was appropriate (item 8). On balance,
the study was judged to be at moderate to high risk of bias.

f) Nakano et al., 2021 [8]. Although the diagnostic criteria were clearly specified (items 1, 4),
the appraisal is dominated by the critically small sample of three PFAPA and four
recurrent-tonsillitis patients, which limits the detail and representativeness of the group description (item 2 unclear) and the reliability of the diagnostic assignment (item 3 unclear). Confounding is a serious problem because the substantial age imbalance between groups
(5.2 versus 11.5 years) was neither identified nor controlled, and the groups were not age-matched
(items 5 and 6 not met). Biomarker measurement is uncertain because interferon-γ was reported in IU/mL, which is incompatible with the units used elsewhere (item 7 unclear). Although Welch's t-test is a defensible choice, distributional assumptions cannot be verified. The statistical power
is negligible at this sample size, rendering the analysis uninformative (item 8 unclear). The study was judged to be at high risk of bias, and its findings are hypothesis-generating only.

g) Takeuchi et al., 2019 [5]. The largest and most mature cohort satisfied most items, including clearly defined criteria, a detailed 18-year single-center description, a valid diagnostic assignment incorporating genetic exclusion of familial Mediterranean fever, and identification of relevant confounders (items 1–5). Confounding control is rated unclear (item 6) because the healthy comparators were adults rather than age-matched children, which introduces indirectness.
In contrast, biomarker measurement by multiplex assay and quantitative CD64 flow cytometry was valid and reliable (item 7). The statistical item is rated unclear (item 8) because several cytokines are summarised as mean ± standard deviation, even though the standard deviations exceed the means, indicating skewed distributions for which median and interquartile range reporting would have been more appropriate. The study was judged to be at low to moderate risk of bias.

5. Summary

Across the seven studies, methodological quality ranged from low (Kapustova et al. [1]) to high (Nakano et al. [8]), with the remainder at moderate or low-to-moderate risk. The recurrent threats to validity were the absence of age-matched healthy pediatric comparators, incomplete control of confounding by age and by sampling time, and uncertain biomarker-measurement validity arising from incompatible assay units and probable transcription or unit errors.
The two studies confined to within-patient flare-versus-interval comparisons were strongest
in controlling for confounding but, by design, provided no information on distinguishing PFAPA from infection or other autoinflammatory diseases [1,2].

These appraisals fed directly into the GRADE evaluation, contributing
to the downgrading for risk of bias. Most heavily, the downgrading was for biomarkers whose supporting evidence rests on the smallest or most methodologically limited cohorts, notably interferon-γ, where one of the three contributing cohorts is at high risk of bias [8].

In addition, the QUADAS-2 appraisal of the three diagnostic-accuracy studies
(all at high risk of bias in patient selection and index test; Supplementary File S4) informed
the downgrade of risk of bias for the interferon-γ discrimination outcome.

6. References:

1.	Kapustova, L.; Banovcin, P.; Bobcakova, A.; Jurkova Malicherova, E.; Kapustova, D.; Petrovicova, O.; Slenker, B.; Markocsy, A.; Oleksak, F.; Vorcakova, K.; et al. The Use of Ketotifen as Long-Term Anti-Inflammatory Prophylaxis in Children with PFAPA Syndrome. Front Immunol 2023, 14, 1302875, doi:10.3389/fimmu.2023.1302875.

2.	Lazea, C.; Damian, L.; Vulturar, R.; Lazar, C. PFAPA Syndrome: Clinical, Laboratory and Therapeutic Features in a Single-Centre Cohort. Int J Gen Med 2022, 15, 6871–6880, doi:10.2147/IJGM.S373942.

3.	Onur, H.; Onur, A.R. Diagnostic Performance of Routine Blood Parameters in Periodic Fever, Aphthous Stomatitis, Pharyngitis, and Adenitis Syndrome. Journal of Clinical Laboratory Analysis 2023, 37, e24934, doi:10.1002/jcla.24934.

4.	Zhu, X.; Yang, Z.; Huang, Y.; Luo, Y.; Yang, J.; He, T. Cytokine Profiles and Laboratory Parameters as Indicators to Distinguish Children with PFAPA and Bacterial Infection. Front. Immunol. 2026, 17, doi:10.3389/fimmu.2026.1683221.

5.	Takeuchi, Y.; Shigemura, T.; Kobayashi, N.; Nagumo, H.; Furumoto, M.; Ogasawara, K.; Fujii, H.; Takizawa, M.; Soga, T.; Matoba, H.; et al. Clinical Features and New Diagnostic Criteria for the Syndrome of Periodic Fever, Aphthous Stomatitis, Pharyngitis, and Cervical Adenitis. International Journal of Rheumatic Diseases 2019, 22, 1489–1497, doi:10.1111/1756-185X.13610.

6.	Pf, W.; Aw, R.; Me, W.; S, M.; Jj, D.; Jb, R.; Mm, L.; Ja, S.; Pm, B. QUADAS-2: A Revised Tool for the Quality Assessment of Diagnostic Accuracy Studies. Annals of internal medicine 2011, 155, doi:10.7326/0003-4819-155-8-201110180-00009.

7.	Guan, C.; Zhou, Q.; Sun, J.; Ying, W.; Hui, X.; Hou, J.; Liu, L.; Wang, C.; Liu, L.; Sun, B.; et al. The Etiological Spectrum of Pediatric FUO and Clinical Management of PFAPA/SURF: A Ten-Year Retrospective Study. Front. Immunol. 2026, 17, doi:10.3389/fimmu.2026.1791904.

8.	Nakano, S.; Kondo, E.; Iwasaki, H.; Akizuki, H.; Matsuda, K.; Azuma, T.; Sato, G.; Kitamura, Y.; Abe, K.; Takeda, N. Differential Cytokine Profiles in Pediatric Patients with PFAPA Syndrome and Recurrent Tonsillitis. J Med Invest 2021, 68, 38–41, doi:10.2152/jmi.68.38.
