# Supplementary material for: Inflammatory biomarkers in pediatric PFAPA syndrome: a systematic review
Source: Front Pediatr. 2026 Sep 3;14:1934055. doi: 10.3389/fped.2026.1934055 (PMC13582404; doi:10.3389/fped.2026.1934055)
Supplement: Supplementary file 3 [file Supplementaryfile3.docx]

**Supplementary File S3. GRADE Evidence Profile.**

1. GRADE Evidence Profile

The certainty of evidence was rated separately for two questions: change during
a flare versus the asymptomatic interval (Question A) and discrimination of PFAPA from infection (Question B). Most biomarkers perform very differently for these two purposes.
A third question, the discrimination of PFAPA from other autoinflammatory diseases (Question C), is not formally graded in the table below because only two cohorts addressed
it [1,2] and neither provided gradable analyte-level diagnostic metrics; the evidence
for Question C is therefore summarised narratively in Section 3.5 and judged, overall,
to be of very low certainty, with no biomarker demonstrating discriminatory value.

| Biomarker | No. of studies (A/B) | Direction of effect | Consistency (A/B) | Risk of Bias | Precision | Overall Certainty (A/B) | Clinical Comment |
| --- | --- | --- | --- | --- | --- | --- | --- |
| WBC | 3/4 | ↑ flare (A); unchanged vs infection (B) | High/High | Moderate | Moderate | Moderate/Low | Flare rise confirmed in three paired cohorts; comparable to infection in four cohorts including Guan’s direct multi-infection comparison; no discriminatory value [1–7] |
| Neutrophils | 3/3 | ↑ flare (A); unchanged vs infection (B) | High/High | Moderate | Moderate | Moderate/Very Low | Marked flare neutrophilia in three paired cohorts; non-specific vs infection; CD64 a more promising neutrophil parameter [1–6] |
| Lymphocytes | 3/- | ↓ flare (A) | Consistent direction (1 of 3 non-significant) | Moderate | Moderate | Low-Moderate/No Data | Relative flare lymphopenia with interval lymphocytosis, concordant across three cohorts including one large (N=135); phase-related, non-discriminating [1,4–6] |
| CRP | 4/4 | ↑↑ flare (A); unchanged or higher in PFAPA vs infection (B) | Very High/Moderate | Moderate | Good (large N) | Moderate-High/Low | ~53-fold flare rise, normalises in interval; in Guan, PFAPA CRP numerically higher than bacterial/viral infection (50.5 vs 26.6/23.5 mg/L), corroborating Onur; a raised CRP cannot favour infection [1–7] |
| SAA | 2/2 | ↑↑↑ flare (A); unchanged vs infection (B) | High/Moderate | Moderate | Moderate | Moderate/Low | Largest dynamic range of any activity marker; in Guan the highest SAA occurred in mycoplasma infection, not PFAPA; not discriminating [1–4] |
| Calprotectin (Serum) | 1/- | ↑ in interval (paradoxical) | Single Study | Moderate | Very Low | Very Low/No Data | Possible subclinical inter-episode inflammation; requires replication [4] |
| IL-6 | 4/3 | ↑ flare (A); unchanged vs infection (B) | Moderate/Very High – no difference | Moderate | Moderate | Low/Low | Sensitive but wholly non-specific; identical in PFAPA and infection [1–4,7] |
| IL-1β | 1/- | Undetectable in serum (no change) | Not assessable | Moderate-Low | Low (Single Study, Adult Controls) | Low/No Data | Inflammasome paradox; not a useful serum marker; IL-18 preferred [2] |
| IL-18 | 1/- | ↑ flare vs healthy | Not assessable | Moderate-Low | Low (Single Study, Adult Controls) | Low/No Data | Promising inflammasome surrogate; needs paediatric validation [2] |
| TNF-α | 2/1 | Modest skewed ↑ (A); ≈0 / no difference (B) | Inconsistent | Moderate | Very Low | Very Low/Very Low | Quantitative flare IQR 2.50–21.82 pg/mL in Guan (lower quartile at assay floor); near-undetectable in Takeuchi; no separation from infection [1–3] |
| IFN-γ | -/3 | ↑ vs infection (B) | No data/High | No data/ High† | Moderate | No data/Low | Most promising discriminating marker; IFN-γ/IL-6 ratio AUC 0.79 and composite model AUC 0.95; limited by small N and unit heterogeneity; QUADAS-2 rated the contributing accuracy studies (Onur, Zhu, Takeuchi) at high risk of bias (two-gate design, in-sample-derived thresholds), so the (B) estimates are derivation-stage (Supplementary File S4) [2,3,7] |

*Citation numbers in the right-hand column refer to the reference list of this Supplementary File S3 ([1]–[7] below); the A/B counts denote the subset providing a flare-vs-interval (A) or PFAPA-vs-infection (B) contrast for that marker. †For the PFAPA vs. infection (B) contrast, the risk-of-bias rating incorporates the QUADAS-2 appraisal of the diagnostic-accuracy contributors (Onur, Zhu, Takeuchi), all at high risk of bias in patient selection and index test (Supplementary File S4).*

2. Summary Hierarchy of Evidence:

When the three clinical questions are considered together, the strongest evidence supports CRP and SAA as markers of disease activity, with the white-cell and neutrophil counts as adequate but redundant adjuncts. For the more demanding task of distinguishing PFAPA from infection, interferon-γ and the IFN-γ/IL-6 ratio carry the highest certainty among the candidates, though this remains low because the supporting accuracy estimates come from studies at high risk of bias on QUADAS-2 (Supplementary File S4); interleukin-18, neutrophil CD64, and a normal procalcitonin remain promising but inadequately validated candidates. Interleukin-6, interleukin-1β, and TNF-α have no demonstrated discriminating value,
and the serum calprotectin paradox remains an interesting but unconfirmed single-study observation.

For differentiating PFAPA from other autoinflammatory diseases, the certainty
of evidence is very low, and no biomarker has demonstrated discriminating value; thus, differentiation rests on clinical phenotype and genetic exclusion rather than on any analyte identified in these cohorts.

References:

1.	Guan, C.; Zhou, Q.; Sun, J.; Ying, W.; Hui, X.; Hou, J.; Liu, L.; Wang, C.; Liu, L.; Sun, B.; et al. The Etiological Spectrum of Pediatric FUO and Clinical Management of PFAPA/SURF: A Ten-Year Retrospective Study. Front. Immunol. **2026**, 17, doi:10.3389/fimmu.2026.1791904.

2.	Takeuchi, Y.; Shigemura, T.; Kobayashi, N.; Nagumo, H.; Furumoto, M.; Ogasawara, K.; Fujii, H.; Takizawa, M.; Soga, T.; Matoba, H.; et al. Clinical Features and New Diagnostic Criteria for the Syndrome of Periodic Fever, Aphthous Stomatitis, Pharyngitis, and Cervical Adenitis. International Journal of Rheumatic Diseases **2019**, 22, 1489–1497, doi:10.1111/1756-185X.13610.

3.	Zhu, X.; Yang, Z.; Huang, Y.; Luo, Y.; Yang, J.; He, T. Cytokine Profiles and Laboratory Parameters as Indicators to Distinguish Children with PFAPA and Bacterial Infection. Front. Immunol. **2026**, 17, doi:10.3389/fimmu.2026.1683221.

4.	Kapustova, L.; Banovcin, P.; Bobcakova, A.; Jurkova Malicherova, E.; Kapustova, D.; Petrovicova, O.; Slenker, B.; Markocsy, A.; Oleksak, F.; Vorcakova, K.; et al. The Use of Ketotifen as Long-Term Anti-Inflammatory Prophylaxis in Children with PFAPA Syndrome. Front Immunol **2023**, 14, 1302875, doi:10.3389/fimmu.2023.1302875.

5.	Onur, H.; Onur, A.R. Diagnostic Performance of Routine Blood Parameters in Periodic Fever, Aphthous Stomatitis, Pharyngitis, and Adenitis Syndrome. Journal of Clinical Laboratory Analysis **2023**, 37, e24934, doi:10.1002/jcla.24934.

6.	Lazea, C.; Damian, L.; Vulturar, R.; Lazar, C. PFAPA Syndrome: Clinical, Laboratory and Therapeutic Features in a Single-Centre Cohort. Int J Gen Med **2022**, 15, 6871–6880, doi:10.2147/IJGM.S373942.

7.	Nakano, S.; Kondo, E.; Iwasaki, H.; Akizuki, H.; Matsuda, K.; Azuma, T.; Sato, G.; Kitamura, Y.; Abe, K.; Takeda, N. Differential Cytokine Profiles in Pediatric Patients with PFAPA Syndrome and Recurrent Tonsillitis. J Med Invest **2021**, 68, 38–41, doi:10.2152/jmi.68.38.
