# Supplementary material for: Inflammatory biomarkers in pediatric PFAPA syndrome: a systematic review
Source: Front Pediatr. 2026 Sep 3;14:1934055. doi: 10.3389/fped.2026.1934055 (PMC13582404; doi:10.3389/fped.2026.1934055)
Supplement: Supplementary file 4 [file Supplementaryfile4.docx]

# Supplementary File S4. QUADAS-2 Appraisal of the Diagnostic-Accuracy Studies

## 1. Rationale

All seven included studies were appraised using the JBI critical appraisal checklists (Supplementary File S2). Three studies additionally reported diagnostic-accuracy estimates for distinguishing PFAPA from a comparator condition (sensitivity, specificity, cut-off values, and/or area under the ROC curve): Onur & Onur [1], Zhu et al. [2], and Takeuchi et al. [3]. Because the JBI tools are not designed to capture the specific biases of diagnostic test-accuracy research, these three studies were additionally appraised with QUADAS-2 [4].

The remaining four studies (Guan et al. [5], Kapustova et al. [6], Lazea et al. [7], Nakano et al. [8]) reported only within-group (flare versus interval) or descriptive between-group comparisons, without accuracy estimates, and were therefore appraised using JBI alone.

## 2. Method

QUADAS-2 was applied across four domains — Patient Selection, Index Test, Reference Standard, and Flow and Timing — each rated for risk of bias, and the first three additionally for concerns regarding applicability, as Low, High, or Unclear.

For every study, the index test was the candidate biomarker(s) or composite criteria evaluated; the reference standard was the clinical diagnosis of PFAPA (modified Marshall and/or Thomas criteria), with culture confirmation for the streptococcal/bacterial comparators where applicable; and the target condition was PFAPA. Two reviewers (P.T., S.K.) independently assessed each study and resolved disagreements by consensus.

## 3. Summary

Figure 1: QUADAS-2 risk-of-bias and applicability summary

Table 1: QUADAS-2 judgments by study and domain

| **Study** | **PS (RoB)** | **IT (RoB)** | **RS (RoB)** | **F&T (RoB)** | **PS (App)** | **IT (App)** | **RS (App)** |
| --- | --- | --- | --- | --- | --- | --- | --- |
| Onur & Onur [1] | High | High | Unclear | Unclear | Low | Low | Low |
| Zhu et al. [2] | High | High | Unclear | High | Low | Low | Low |
| Takeuchi et al. [3] | High | High | High | High | High | High | Unclear |

Footnote: RoB = risk of bias; App = applicability concerns; PS = patient selection;
IT = index test; RS = reference standard; F&T = flow and timing.

## 4. Domain-level justification

a) Onur & Onur [1]

**Patient selection — High:** a two-gate (case–control–type) design comparing pre-diagnosed PFAPA with pre-diagnosed streptococcal pharyngitis, with exclusion of diagnostically unclear cases, a configuration that inflates apparent accuracy.

**Index test — High:** the discriminating cut-offs (age >49 months, CRP ≤9.8 mg/dL,
ESR ≤18 mm/h, NLR >3.24) were derived from the same ROC analysis in which they were evaluated, with no pre-specification or external validation.

**Reference standard — Unclear:** PFAPA was defined by clinical criteria with no independent gold standard, and the single diagnosing physician had access to laboratory values, raising the possibility of incorporation bias; blinding was not described.

**Flow & timing — Unclear:** timing of sampling relative to diagnosis and blinding of the reference standard were not reported, although all participants received the same work-up.

b) Zhu et al. [2]

**Patient selection — High:** a retrospective two-gate comparison of PFAPA (n=67) with a broad bacterial-infection group (n=160).

**Index test — High:** the cytokine cut-offs (IFN-γ/IL-6 ratio 0.43; composite logistic-regression score 0.38, AUC 0.95) were both derived and tested in the same cohort, without external validation, so the reported accuracy is at high risk of optimistic (overfitting) bias.

**Reference standard — Unclear:** PFAPA diagnosis rested on clinical criteria; blinding
of the reference standard to the research cytokine assays was not described, although these assays were unlikely to have informed routine diagnosis.

**Flow & timing — High:** sampling occurred at different time points in the two groups (median day 2 in PFAPA versus day 6 in bacterial infection), which may independently influence biomarker concentrations, independent of diagnosis.

c) Takeuchi et al. [3]

**Patient selection — High:** a multi-gate design comparing pre-diagnosed PFAPA (n=257) with infectious disease (n=104) and with a genotype-restricted FMF group (MEFV M694I only, n=67).

**Index test — High:** the proposed (Takeuchi) criteria were derived from the same PFAPA cohort and then tested on it, and the threshold (≥4 versus ≥5 supportive items) was selected in-sample, producing circular, optimistic estimates.

**Reference standard — High:** the reference standard (Marshall/Thomas clinical criteria) overlaps substantially with the index criteria themselves, creating strong incorporation/circularity.

**Flow & timing — High:** index tests were applied to varying subsets (cytokines n=94, CD64 n=134, IgD n=199 of 257), i.e., partial and selective verification.

**Applicability — High (patient selection and index test):** the FMF comparator was restricted to a single genotype and may not represent FMF generally; the reported accuracy reflects
a composite clinical-plus-biomarker criteria set rather than any biomarker in isolation,
and only partially matches a biomarker-focused review question.

## 4. Interpretation

Across all three studies, risk of bias was high in the Patient Selection and Index Test domains, driven by two recurring features: two-gate (case–control–type) sampling
and derivation of discriminating thresholds within the same dataset used to evaluate them. Consequently, the headline accuracy estimates in this field, including the composite cytokine model of Zhu et al. (AUC 0.95), the age and CRP thresholds of Onur & Onur (AUC 0.713 and 0.607), and the criteria performance of Takeuchi et al. (93.8% sensitivity, 95.6% specificity versus FMF).

It should be regarded as derivation-stage findings at high risk of optimistic bias,
not as externally validated diagnostic performance. This reinforces the central conclusion
of the review: no inflammatory biomarker has yet been shown, in an appropriately designed and externally validated study, to distinguish PFAPA from infection or from other autoinflammatory diseases.

## References:

1.	Onur, H.; Onur, A.R. Diagnostic Performance of Routine Blood Parameters in Periodic Fever, Aphthous Stomatitis, Pharyngitis, and Adenitis Syndrome. Journal of Clinical Laboratory Analysis 2023, 37, e24934, doi:10.1002/jcla.24934.

2.	Zhu, X.; Yang, Z.; Huang, Y.; Luo, Y.; Yang, J.; He, T. Cytokine Profiles and Laboratory Parameters as Indicators to Distinguish Children with PFAPA and Bacterial Infection. Front. Immunol. 2026, 17, doi:10.3389/fimmu.2026.1683221.

3.	Takeuchi, Y.; Shigemura, T.; Kobayashi, N.; Nagumo, H.; Furumoto, M.; Ogasawara, K.; Fujii, H.; Takizawa, M.; Soga, T.; Matoba, H.; et al. Clinical Features and New Diagnostic Criteria for the Syndrome of Periodic Fever, Aphthous Stomatitis, Pharyngitis, and Cervical Adenitis. International Journal of Rheumatic Diseases 2019, 22, 1489–1497, doi:10.1111/1756-185X.13610.

4.	Whiting, P.F.; Rutjes, A.W.S.; Westwood, M.E.; Mallett, S.; Deeks, J.J.; Reitsma, J.B.; Leeflang, M.M.G.; Sterne, J.A.C.; Bossuyt, P.M.M. QUADAS-2: A Revised Tool for the Quality Assessment of Diagnostic Accuracy Studies. Ann. Intern. Med. 2011, 155, 529–536, Doi:10.7326/0003-4819-155-8-201110180-00009.

5.	Guan, C.; Zhou, Q.; Sun, J.; Ying, W.; Hui, X.; Hou, J.; Liu, L.; Wang, C.; Liu, L.; Sun, B.; et al. The Etiological Spectrum of Pediatric FUO and Clinical Management of PFAPA/SURF: A Ten-Year Retrospective Study. Front. Immunol. 2026, 17, doi:10.3389/fimmu.2026.1791904.

6.	Kapustova, L.; Banovcin, P.; Bobcakova, A.; Jurkova Malicherova, E.; Kapustova, D.; Petrovicova, O.; Slenker, B.; Markocsy, A.; Oleksak, F.; Vorcakova, K.; et al. The Use of Ketotifen as Long-Term Anti-Inflammatory Prophylaxis in Children with PFAPA Syndrome. Front Immunol 2023, 14, 1302875, doi:10.3389/fimmu.2023.1302875.

7.	Lazea, C.; Damian, L.; Vulturar, R.; Lazar, C. PFAPA Syndrome: Clinical, Laboratory and Therapeutic Features in a Single-Centre Cohort. Int J Gen Med 2022, 15, 6871–6880, doi:10.2147/IJGM.S373942.

8.	Nakano, S.; Kondo, E.; Iwasaki, H.; Akizuki, H.; Matsuda, K.; Azuma, T.; Sato, G.; Kitamura, Y.; Abe, K.; Takeda, N. Differential Cytokine Profiles in Pediatric Patients with PFAPA Syndrome and Recurrent Tonsillitis. J Med Invest 2021, 68, 38–41, doi:10.2152/jmi.68.38.
