# Supplementary material for: Inflammatory biomarkers in pediatric PFAPA syndrome: a systematic review
Source: Front Pediatr. 2026 Sep 3;14:1934055. doi: 10.3389/fped.2026.1934055 (PMC13582404; doi:10.3389/fped.2026.1934055)
Supplement: Supplementary file 5 [file Supplementaryfile5.docx]

**Supplementary File S5. Full biomarker comparison tables (all subgroups).**

These are the complete, all-group versions of the abridged main-text Tables
3a-3c. Each row corresponds to one subgroup (disease phase or comparator)
as reported in the primary study. Where a study reported infection comparators within a single cell (Guan et al. [1]), these are shown here as separate rows. Values
are reproduced as originally published (mean±SD or median [IQR]). The abridged one-row-per-study versions appear in the main text (Tables 3a–3c).

1. Hematological parameters

Units: White blood cells (WBC), neutrophils (NEUT), and lymphocytes (LYMPH)
are reported in the original units; ×10⁹/L and ×10³/mm³ are numerically equivalent, and /mm³ = ×10⁶/L. Takeuchi et al. [2] reported neutrophils only as a percentage (segmented + band), not as an absolute count. "Not reported" denotes a parameter
not measured or not provided in the source. Values for Guan et al. [1] are taken from the study's own Supplementary Table S1 and S2 (immune indices by subgroup
and disease phase).

Table S5a. Hematological parameters (all included studies with all subgroups)

| Study | Group | WBC | NEUT | LYMPH | Intra study conclusion |
| --- | --- | --- | --- | --- | --- |
| Zhu et al., 2026 [3] | PFAPA – flare | 10.7 (8.9–13.3) ×10⁹/L | 7.5 (6.1–9.6) ×10⁹/L | Not reported | WBC (p=0.477) and NEUT (p=0.835) non-significant vs bacterial infection |
| Zhu et al., 2026 [3] | Bacterial infection – flare | 11.6 (7.9–16.9) ×10⁹/L | 7.2 (4.8–12.3) ×10⁹/L | Not reported | As above |
| Guan et al., 2026 [1] | PFAPA – flare | 12.35 x 10^9^/L (mean)  4.31 x 10^9^/L (SD)  *Add.:*  *Bacterial infection:*  *11.88 x 10^9^/L (mean); 5.73 x 10^9^/L (SD)*  *Viral infection:*  *8.71 x 10^9^/L (mean); 4.08 x 10^9^/L (SD)*  *Mycoplasma infection:*  *12.08 x 10^9^/L (mean); 7.76 x 10^9^/L (SD)* | 8.46 x 10^9^/L (mean)  3.91 x 10^9^/L (SD) | 2.61 x 10^9^/L (mean)  1.07 x 10^9^/L (SD) | ↑WBC and ↑NEUT at flare vs PFAPA remission period; no difference between SURF flare |
| Guan et al., 2026 [1] | PFAPA – remission period | 7.13 x 10^9^/L (mean)  2.20 x 10^9^/L (SD) | 3.00 x 10^9^/L (mean)  1.68 x 10^9^/L (SD) | 3.38 x 10^9^/L (mean)  1.27 x 10^9^/L (SD) | PFAPA remission period vs non-flare periodic and non-flare non-periodic is non significant |
| Guan et al., 2026 [1] | SURF periodic – flare | 12.97 x 10^9^/L (mean)  4.06 x 10^9^/L (SD) | 8.50 x 10^9^/L (mean)  3.74 x 10^9^/L (SD) | 2.88 x 10^9^/L (mean)  0.96 x 10^9^/L (SD) | Flare values in SURF periodic and non-periodic nearly identical to PFAPA; inter-subgroup differences non-significant |
| Guan et al., 2026 [1] | SURF periodic – non-flare | 7.27 x 10^9^/L (mean)  2.16 x 10^9^/L (SD) | 2.90 x 10^9^/L (mean)  1.55 x 10^9^/L (SD) | 3.63 x 10^9^/L (mean)  1.53 x 10^9^/L (SD) | As above |
| Guan et al., 2026 [1] | SURF non-periodic – flare | 12.96 x 10^9^/L (mean)  6.84 x 10^9^/L (SD) | 7.8 x 10^9^/L (mean)  5.58 x 10^9^/L (SD) | 2.69 x 10^9^/L (mean)  1.23 x 10^9^/L (SD) | Flare values nearly identical to PFAPA; non-significant |
| Guan et al., 2026 [1] | SURF non-periodic – non-flare | 7.31 x 10^9^/L (mean)  1.89 x 10^9^/L (SD) | 3.53 x 10^9^/L (mean)  1.77 x 10^9^/L (SD) | 2.77 x 10^9^/L (mean)  0.53 x 10^9^/L (SD) | As above |
| Kapustova et al., 2023 [4] | PFAPA – flare | 11.95 (8.6–14.8) ×10⁹/L | 6.45 (4.65–9.85) ×10⁹/L | 2.75 (2.12–4.05) ×10⁹/L | Flare vs attack-free period: ↑WBC, ↑NEUT, (all p<0.0001); ↓LYMPH (p=0.0009) |
| Kapustova et al., 2023 [4] | PFAPA – attack-free | 8.05 (6.4–9.5) ×10⁹/L | 3.15 (2.3–4.1) ×10⁹/L | 3.82 (2.8–5.27) ×10⁹/L | ↑LYMPH (p=0.0009) in the interval |
| Onur & Onur, 2023 [5] | PFAPA - flare | 14,040±4,410 /mm³ | 8,820 (1,380–23,170) /mm³ | 2,860 (1,110–5,770) /mm³ | WBC (p=0.231), NEUT (p=0.233), LYMPH (p=0.170) all non-significant vs strep pharyngitis |
| Onur & Onur, 2023 [5] | Strep pharyngitis – flare | 15,130±6,190 /mm³ | 9,500 (1,820–31,820) /mm³ | 2,760 (840–7,600) /mm³ | As above |
| Lazea et al., 2022 [6] | PFAPA – episode | 14,839 (8,600–22,600) /mm³ | 9,977 (3,732–20,540) /mm³ | 3,683 (1,940–6,810) /mm³ | PFAPA flare vs inter-episode: ↑WBC (p<0.0001), ↑NEUT (p=0.0001), ↑monocytes (p=0.0058); ↓LYMPH non-significant (p=0.0707) |
| Lazea et al., 2022 [6] | PFAPA – inter-episode | 8,922 (5,000–138,000*) /mm³ | 3,386 (1,420–7,064) /mm³ | 4,568 (1,015–9,080) /mm³ | Inflammatory normalization; *upper WBC bound 138,000 likely a typographical error in the source, reported verbatim |
| Nakano et al., 2021 [7] | PFAPA – flare | 10,977 /mm³ (mean, no SD) | Not reported | Not reported | No significant difference in WBC vs recurrent tonsillitis |
| Nakano et al., 2021 [7] | Recurrent tonsillitis – flare | 13,015 /mm³ (mean, no SD) | Not reported | Not reported | As above |
| Takeuchi et al., 2019 [2] | PFAPA – flare | 12,500±4,700 /μL | 66.1±12.7% Seg + 3.7±4.8% Band (%, not absolute) | Not reported | Marked leucocytosis at flare onset; neutrophils reported only as percentage; no within-study flare/remission contrast in main text |
| Takeuchi et al., 2019 [2] | Bacterial infection – flare | Not reported | Not reported | Not reported | Not applicable |
| Takeuchi et al., 2019 [2] | Healthy controls (adults) | Not reported | Not reported | Not reported | Not applicable |

*Abbreviations: Band – Band Neutrophils; L – Liter; LYMPH – Lymphocytes; mm^3^ – Cubic Millimeter; NEUT – Neutrophils; PFAPA – Periodic Fever, Aphthous Stomatitis, Pharyngitis and Adenitis; SD – Standard Deviation; Seg – Segmental Neutrophils; SURF – Syndrome of Undifferentiated Recurrent Fever; WBC – White Blood Cells.*

2. Inflammatory markers and cytokines (Table S5b and Table S5c)

CRP is reported in mg/L in Kapustova et al. [4] and Zhu et al. [3], and in mg/dL in Lazea et al. [6], Nakano et al. [7], Onur & Onur [5], and Takeuchi et al. [2] (1 mg/dL = 10 mg/L); values are presented as published. SAA in mg/L equals μg/mL. IL-6 in pg/mL equals ng/L. IFN-γ was reported in IU/mL by Nakano et al. [7], which is not directly convertible to the pg/mL used in Takeuchi et al. [2] and Zhu et al. [3]. Calprotectin was measured in serum only, not in stool.

Table S5b: Acute phase reactants (CRP, SAA, serum calprotectin) and proteins (all included studies with subgroups):

| Study | Group | CRP | SAA | Serum calprotectin | Intra study conclusion |
| --- | --- | --- | --- | --- | --- |
| Zhu et al., 2026 [3] | PFAPA – flare | 41.1 (21.4-71.2) mg/L | 113.2 (53.15-350.2) mg/L | NR | SAA and CRP non-significant |
| Zhu et al., 2026 [3] | Bacterial infection – flare | 49.4 (21.8-84.8) mg/L | 116.5 (54.71–414.5) mg/L | NR | Not applicable |
| Guan et al., 2026 [1] | PFAPA – flare | 50.54 mg/L (mean)  35.28 mg/L (SD)  *Add.:*  *Bacterial infection:*  *26.56 mg/L (mean); 23.39 mg/L (SD)*  *Viral infection:*  *23.45 mg/L (mean); 26.35 mg/L (SD)*  *Mycoplasma infection:*  *35.86 mg/L (mean); 36.28 mg/L (SD)* | 238.14 mg/L (mean)  197.67 mg/L (SD)  *Add.*  *Bacterial infection:*  *86.09 mg/L (mean); 72.12 mg/L (SD)*  *Viral infection:*  *70.18 mg/L (mean); 76.99 mg/L (SD)*  *Mycoplasma infection:*  *303.78 mg/L (mean); 230.08 mg/L (SD)* | NR | ↑CRP at flare; normalization between episodes (criteria require CRP <0.2 mg/L in remission)  PFAPA-vs-infection comparison: PFAPA CRP higher than bacterial (26.56) and viral (23.45) infection; SAA values are lower in viral/bacterial than in mycoplasma/SURF /PFAPA flare values |
| Guan et al., 2026 [1] | PFAPA – remission period | NR | NR | NR | Not applicable |
| Guan et al., 2026 [1] | SURF periodic – flare | 41.03 mg/L (mean)  32.52 mg/L (SD) | 320.98 mg/L (mean)  179.13 mg/L (SD) | NR | Higher mean SAA vs other subgroups |
| Guan et al., 2026 [1] | SURF periodic – non-flare | Elevated | NR | NR | No significant inter-subgroup difference |
| Guan et al., 2026 [1] | SURF non-periodic – flare | 46.13 mg/L (mean)  42.29 mg/L (SD) | 228.31 mg/L (mean)  173.75 mg/L (SD) | NR | No significant inter-subgroup difference |
| Guan et al., 2026 [1] | SURF non-periodic – non-flare | NR | NR | NR | No significant inter-subgroup difference |
| Kapustova et al., 2023 [4] | PFAPA – flare | 50 (17.1–120) mg/L | 132 (36.65–278.25) mg/L | 3.64 (2.94–6.28) μg/mL | PFAPA flare vs interval: ↑↑CRP (p<0.0001), ↑↑SAA (p<0.0001); calprotectin lower in flare than interval (see below) |
| Kapustova et al., 2023 [4] | PFAPA – attack-free | 0.95 (0.3–2.3) mg/L | 4 (3.4–7.43) mg/L | 5.92 (3.11–12.8) μg/mL | Serum calprotectin HIGHER in the interval than in flare (p=0.036); interpreted by the authors as subclinical inter-episode inflammation |
| Onur & Onur, 2023 [5] | PFAPA - flare | 32 (9.26–225) mg/dL¹ | NR | NR | ↑CRP (p=0.026) vs strep pharyngitis |
| Onur & Onur, 2023 [5] | Strep pharyngitis – flare | 27.13 (0.21–133.9) mg/dL¹ | NR | NR | Lower CRP than PFAPA |
| Lazea et al., 2022 [6] | PFAPA – episode | 7.9 (1.6–15.8) mg/dL | NR | NR | ↑CRP (p<0.0001) vs inter-episode |
| Lazea et al., 2022 [6] | PFAPA – inter-episode | 0.5 (0.2–2.3) mg/dL | NR | NR | Normalization of acute-phase reactant |
| Nakano et al., 2021 [7] | PFAPA – flare | 3.13 mg/dL (mean) | NR | NR | CRP non-significant difference between subgroups |
| Nakano et al., 2021 [7] | Recurrent tonsillitis – flare | 4.48 mg/dL (mean) | NR | NR | As above |
| Takeuchi et al., 2019 [2] | PFAPA – flare | 6.7±4.6 mg/dL | 669.2±449.8 μg/mL | NR | ↑↑SAA vs controls and bacterial infection |
| Takeuchi et al., 2019 [2] | Bacterial infection – flare | NR | NR | NR | Not applicable |
| Takeuchi et al., 2019 [2] | Healthy controls (adults) | NR | NR | NR | Not applicable |

*Footnote: ¹ Reviewer note: Onur & Onur [54] label CRP as mg/dL, yielding a PFAPA median of 32 mg/dL (= 320 mg/L) and an upper range of 225 mg/dL (= 2,250 mg/L). These values are an order of magnitude higher than those of every other cohort (Zhu et al. [65] 41.1 mg/L; Kapustova et al. [58] 50 mg/L), and a concentration of 2,250 mg/L is physiologically implausible, strongly suggesting that the unit label is erroneous and that the true unit is mg/L. The values are reported here verbatim but should be interpreted with caution. Abbreviations: CRP – C-reactive Protein; mg/L – Milligrams per Liter; mg/dL – Milligrams per Milliliter; μg/mL – Micrograms per Milliliter; NR = not reported; SAA – Serum Amyloid A.*

Table S5c: Acute phase reactants and proteins (cytokines and interferons) (all included studies with all subgroups):

| Study | Group | IL-6 | IL-1β | IL-18 | TNF-α | IFN-γ | Intra study conclusion |
| --- | --- | --- | --- | --- | --- | --- | --- |
| Zhu et al., 2026 [3] | PFAPA – flare | 25.1 (2.8-65) pg/mL | NR | NR | 2.4 (0.9-3.1) pg/mL | 16.3 (5.6-40.8) pg/mL | ↑↑IFN-γ (p<0.0001), ↑IFN-γ/IL-6 ratio (0.7 vs 0.2; AUC 0.79); IL-6 non-significant |
| Zhu et al., 2026 [3] | Bacterial infection – flare | 27.6 (13.2–66.8) pg/mL | NR | NR | 1.9 (0.7–3.2) pg/mL | 3.2 (1.7–7.7) pg/mL | IFN-γ much lower than PFAPA; IL-6 comparable |
| Guan et al., 2026 [1] | PFAPA – flare | IQR: 20.15 -114.52 pg/mL | NR | NR | IQR: 2.50-21.82 pg/mL | NR | ↑IL-6, ↑TNF-α at flare; normalization between episodes; IL-6 higher in PFAPA vs SURF subgroups, but differences non-significant |
| Guan et al., 2026 [1] | PFAPA – remission period | NR | NR | NR | NR | NR | Not applicable |
| Guan et al., 2026 [1] | SURF periodic – flare | IQR: 11.8-59.3 pg/mL | NR | NR | IQR: 2.50-11.97 pg/mL | NR | IL-6 and TNF-α lower than PFAPA; no significant inter-subgroup difference |
| Guan et al., 2026 [1] | SURF periodic – non-flare | NR | NR | NR | NR | NR | Not applicable |
| Guan et al., 2026 [1] | SURF non-periodic – flare | IQR: 5.70-91.1 pg/mL | NR | NR | IQR: 2.50-18.50 pg/mL | NR | Widest IL-6 range; overlapping with PFAPA; non-significant |
| Guan et al., 2026 [1] | SURF non-periodic – non-flare | NR | NR | NR | NR | NR | Not applicable |
| Kapustova et al., 2023 [4] | PFAPA – flare | 6.94 (1.87–20.64) ng/L | NR | NR | NR | NR | PFAPA flare vs interval: ↑IL-6 (p<0.0001) |
| Kapustova et al., 2023 [4] | PFAPA – attack-free | 1.52 (1.5–2.89) ng/L | NR | NR | NR | NR | As above |
| Onur & Onur, 2023 [5] | PFAPA - flare | NR | NR | NR | NR | NR | Not applicable |
| Onur & Onur, 2023 [5] | Strep pharyngitis – flare | NR | NR | NR | NR | NR | Not applicable |
| Lazea et al., 2022 [6] | PFAPA – episode | NR | NR | NR | NR | NR | Not applicable |
| Lazea et al., 2022 [6] | PFAPA – inter-episode | NR | NR | NR | NR | NR | Not applicable |
| Nakano et al., 2021 [7] | PFAPA – flare | 33.8±5.6 pg/mL | NR | NR | NR | 319.0±18.3 IU/mL | ↑↑IFN-γ (319 vs 1.02 IU/mL, p<0.01) vs tonsillitis; IL-6 non-significant |
| Nakano et al., 2021 [7] | Recurrent tonsillitis – flare | 46.4±18.8 pg/mL | NR | NR | NR | 1.02±0.72 IU/mL | IFN-γ within normal range (contrast with PFAPA) |
| Takeuchi et al., 2019 [2] | PFAPA – flare | 35.0±89.5 pg/mL | <5 pg/mL (below detection) | 477.1±226.2 pg/mL | 0.7±2.1 pg/mL | 17.8±21.1 pg/mL | ↑IFN-γ vs controls and bacterial infection; IL-1β undetectable; IL-18 elevated vs healthy |
| Takeuchi et al., 2019 [2] | Bacterial infection – flare | 32.5±20.8 pg/mL | <5 pg/mL | NR | <5 pg/mL | <5 pg/mL | IL-6 comparable to PFAPA; IFN-γ <5 pg/mL (vs 17.8 in PFAPA); TNF-α undetectable |
| Takeuchi et al., 2019 [2] | Healthy controls (adults) | <5 pg/mL | <5 pg/mL | 166.7±140.2 | <5 pg/mL | <5 pg/mL | IL-18 lower than in PFAPA; other inflammatory cytokines at or below detection |

*Abbreviations: AUC – Area Under the Curve; IFN-γ – Interferon Gamma; IL-1β – Interleukin 1 Beta; IL-6 – Interleukin 6; IL-18 – Interleukin 18; IQR – Interquartile Range; IU/mL – International Units per Milliliter; NR – Not Reported; p – P-Value; PFAPA – Periodic Fever, Aphthous Stomatitis, Pharyngitis, And Adenitis; pg/mL – Picograms per Milliliter; SURF – Syndrome Of Undifferentiated Recurrent Fever; TNF-α – Tumor Necrosis Factor Alpha.*

References:

1.	Guan, C.; Zhou, Q.; Sun, J.; Ying, W.; Hui, X.; Hou, J.; Liu, L.; Wang, C.; Liu, L.; Sun, B.; et al. The Etiological Spectrum of Pediatric FUO and Clinical Management of PFAPA/SURF: A Ten-Year Retrospective Study. Front. Immunol. 2026, 17, doi:10.3389/fimmu.2026.1791904.

2.	Takeuchi, Y.; Shigemura, T.; Kobayashi, N.; Nagumo, H.; Furumoto, M.; Ogasawara, K.; Fujii, H.; Takizawa, M.; Soga, T.; Matoba, H.; et al. Clinical Features and New Diagnostic Criteria for the Syndrome of Periodic Fever, Aphthous Stomatitis, Pharyngitis, and Cervical Adenitis. International Journal of Rheumatic Diseases 2019, 22, 1489–1497, doi:10.1111/1756-185X.13610.

3.	Zhu, X.; Yang, Z.; Huang, Y.; Luo, Y.; Yang, J.; He, T. Cytokine Profiles and Laboratory Parameters as Indicators to Distinguish Children with PFAPA and Bacterial Infection. Front. Immunol. 2026, 17, doi:10.3389/fimmu.2026.1683221.

4.	Kapustova, L.; Banovcin, P.; Bobcakova, A.; Jurkova Malicherova, E.; Kapustova, D.; Petrovicova, O.; Slenker, B.; Markocsy, A.; Oleksak, F.; Vorcakova, K.; et al. The Use of Ketotifen as Long-Term Anti-Inflammatory Prophylaxis in Children with PFAPA Syndrome. Front Immunol 2023, 14, 1302875, doi:10.3389/fimmu.2023.1302875.

5.	Onur, H.; Onur, A.R. Diagnostic Performance of Routine Blood Parameters in Periodic Fever, Aphthous Stomatitis, Pharyngitis, and Adenitis Syndrome. Journal of Clinical Laboratory Analysis 2023, 37, e24934, doi:10.1002/jcla.24934.

6.	Lazea, C.; Damian, L.; Vulturar, R.; Lazar, C. PFAPA Syndrome: Clinical, Laboratory and Therapeutic Features in a Single-Centre Cohort. Int J Gen Med 2022, 15, 6871–6880, doi:10.2147/IJGM.S373942.

7.	Nakano, S.; Kondo, E.; Iwasaki, H.; Akizuki, H.; Matsuda, K.; Azuma, T.; Sato, G.; Kitamura, Y.; Abe, K.; Takeda, N. Differential Cytokine Profiles in Pediatric Patients with PFAPA Syndrome and Recurrent Tonsillitis. J Med Invest 2021, 68, 38–41, doi:10.2152/jmi.68.38.
