# Supplementary material for: Inflammatory biomarkers in pediatric PFAPA syndrome: a systematic review
Source: Front Pediatr. 2026 Sep 3;14:1934055. doi: 10.3389/fped.2026.1934055 (PMC13582404; doi:10.3389/fped.2026.1934055)
Supplement: Supplementary file 6 [file Table1.docx]

**Supplementary Table S1. PRISMA 2020 Checklist**

*Inflammatory Biomarkers in Pediatric PFAPA Syndrome: A Systematic Review (P. Treichel, S. Kołtan, J. Styczyński).*

Checklist adapted from: Page MJ et al. The PRISMA 2020 statement: an updated guideline for reporting systematic reviews. BMJ 2021;372:n71 (CC BY 4.0). “Location” refers to manuscript sections, figures, tables, and supplementary files. Status labels (Reported / Partially reported / Not reported / Not applicable) reflect the reviewer’s appraisal of this manuscript.

| **Section and Topic** | **Item #** | **Checklist item** | **Location where item is reported** |
| --- | --- | --- | --- |
| **TITLE** | | | |
| **Title** | **1** | Identify the report as a systematic review. | **Reported** Title: “Inflammatory Biomarkers in Pediatric PFAPA Syndrome: A Systematic Review” explicitly identifies the report as a systematic review. |
| **ABSTRACT** | | | |
| **Abstract** | **2** | See the PRISMA 2020 for Abstracts checklist. | **Reported** Abstract: structured as Background, Methods, Results, Conclusions; states objectives, eligibility, sources (PubMed/Scopus), synthesis method, main results, certainty (GRADE) and conclusions. Keywords provided. |
| **INTRODUCTION** | | | |
| **Rationale** | **3** | Describe the rationale for the review in the context of existing knowledge. | **Reported** Section 1 (Introduction), including the knowledge-gap paragraph and Abstract (Background). |
| **Objectives** | **4** | Provide an explicit statement of the objective(s) or question(s) the review addresses. | **Reported** Abstract (aim); Section 2.1 (Study design and primary aim); Supplementary File S1 (PECO question). |
| **METHODS** | | | |
| **Eligibility criteria** | **5** | Specify the inclusion and exclusion criteria for the review and how studies were grouped for the syntheses. | **Reported** Section 2.3 (2.3.1 inclusion; 2.3.2 exclusion). Grouping by the three review questions (activity; PFAPA vs infection; PFAPA vs other autoinflammatory disease) in Section 2.8.  Supplementary File S1. |
| **Information sources** | **6** | Specify all databases, registers, websites, organisations, reference lists and other sources searched or consulted to identify studies. Specify the date when each source was last searched or consulted. | **Reported** Section 2.4: PubMed/MEDLINE and Scopus; last search 07 June 2026; date range 1 Jan 2018–07 Jun 2026; reference-list checking, citation tracking and grey literature explicitly NOT used. Supplementary Table S2. |
| **Search strategy** | **7** | Present the full search strategies for all databases, registers and websites, including any filters and limits used. | **Reported** Supplementary Table S2: full PubMed and Scopus strings (three query blocks each), record counts, date range, last-search date, filters (none applied); summarised in Section 2.4. |
| **Selection process** | **8** | Specify the methods used to decide whether a study met the inclusion criteria of the review, including how many reviewers screened each record and each report retrieved, whether they worked independently, and if applicable, details of automation tools used in the process. | **Reported** Section 2.5: two independent reviewers screened titles/abstracts and full texts; Rayyan used for de-duplication and screening; disagreements resolved by consensus or a third reviewer. |
| **Data collection process** | **9** | Specify the methods used to collect data from reports, including how many reviewers collected data from each report, whether they worked independently, any processes for obtaining or confirming data from study investigators, and if applicable, details of automation tools used in the process. | **Reported** Section 2.6: standardized extraction form; two reviewers extracted independently; values sourced from full text/PMC/publisher records and supplementary files; discrepancies resolved by discussion. No contact with original investigators was reported. |
| **Data items** | **10a** | List and define all outcomes for which data were sought. Specify whether all results that were compatible with each outcome domain in each study were sought (e.g. for all measures, time points, analyses), and if not, the methods used to decide which results to collect. | **Reported** Sections 2.3 and 2.6: eleven predefined biomarkers (WBC, neutrophils, lymphocytes, CRP, SAA, calprotectin, IL-6, IL-1β, IL-18, TNF-α, IFN-γ), sought by disease phase (flare / interval) and by comparator (infection, SURF, other). |
| **Data items** | **10b** | List and define all other variables for which data were sought (e.g. participant and intervention characteristics, funding sources). Describe any assumptions made about any missing or unclear information. | **Reported** Section 2.6: all other variables sought are listed (author, year, DOI/PMID, country, design, n [PFAPA/comparator], diagnostic criteria, age, disease phase, comparator) and the handling of missing/unclear data is described (coded “Not reported”, NR). Funding of the included primary studies was, by design, outside the predefined extraction set. |
| **Study risk of bias assessment** | **11** | Specify the methods used to assess risk of bias in the included studies, including details of the tool(s) used, how many reviewers assessed each study and whether they worked independently, and if applicable, details of automation tools used in the process. | **Reported** Section 2.7: JBI Critical Appraisal Checklist for Analytical Cross-Sectional Studies, applied uniformly to all 7 cohorts (cohort items additionally considered for two paired-sampling studies) and QUADAS-2 for the three diagnostic-accuracy studies; two reviewers independently (P.T., S.K.); see Section 2.7 and Supplementary Files S2 and S4. |
| **Effect measures** | **12** | Specify for each outcome the effect measure(s) (e.g. risk ratio, mean difference) used in the synthesis or presentation of results. | **Reported** Section 2.8: the presentation format (effect measure) is specified for the narrative synthesis: continuous biomarker values as originally published (mean ± SD or median [IQR/range]) with the direction of change between phases/comparators; diagnostic-accuracy metrics (e.g. AUC) where primary studies reported them. No pooled estimate was used because no meta-analysis was performed. |
| **Synthesis methods** | **13a** | Describe the processes used to decide which studies were eligible for each synthesis (e.g. tabulating the study intervention characteristics and comparing against the planned groups for each synthesis). | **Reported** Section 2.8: each study treated as an independent unit; syntheses structured around three predefined questions (disease activity; PFAPA vs infection; PFAPA vs other autoinflammatory disease). |
| **Synthesis methods** | **13b** | Describe any methods required to prepare the data for presentation or synthesis, such as handling of missing summary statistics, or data conversions. | **Reported** Sections 2.6 and 2.8: data reported as originally published; no unit conversion attempted (incompatible IFN-γ units, IU/mL vs pg/mL, noted as a barrier to pooling); missing parameters coded “Not reported”. |
| **Synthesis methods** | **13c** | Describe any methods used to tabulate or visually display results of individual studies and syntheses. | **Reported** Sections 2.8 and 3: structured summary tables: study characteristics (Table 2) and hematological / inflammatory-cytokine / additional-derived biomarker tables (Tables 3a–3d); full between-group versions in Supplementary File S5. |
| **Synthesis methods** | **13d** | Describe any methods used to synthesize results and provide a rationale for the choice(s). If meta-analysis was performed, describe the model(s), method(s) to identify the presence and extent of statistical heterogeneity, and software package(s) used. | **Reported** Section 2.8: structured narrative (qualitative) synthesis; meta-analysis judged inappropriate owing to heterogeneity in comparator groups, assay platforms, units and reporting formats (rationale given). No model/software for pooling, as no meta-analysis was conducted. |
| **Synthesis methods** | **13e** | Describe any methods used to explore possible causes of heterogeneity among study results (e.g. subgroup analysis, meta-regression). | **Reported** Section 2.8 (applied in Sections 3.1 and 4): causes of heterogeneity were explored narratively (comparator type/severity, assay platforms and units, variation in diagnostic criteria). Subgroup analysis and meta-regression were not applicable because no meta-analysis was undertaken. |
| **Synthesis methods** | **13f** | Describe any sensitivity analyses conducted to assess robustness of the synthesized results. | **Not applicable**  No quantitative synthesis was performed, so no review-level sensitivity analyses were possible (Section 2.8). (A sensitivity analysis within one primary study, Zhu et al., is reported descriptively in the Discussion.) |
| **Reporting bias assessment** | **14** | Describe any methods used to assess risk of bias due to missing results in a synthesis (arising from reporting biases). | **Reported** Section 2.8 and Supplementary File S3: risk of bias from missing results was assessed within GRADE through the publication-bias domain. A funnel plot / statistical small-study test was not applicable in the absence of meta-analysis. |
| **Certainty assessment** | **15** | Describe any methods used to assess certainty (or confidence) in the body of evidence for an outcome. | **Reported** Section 2.8: GRADE applied per biomarker for Question A (activity) and Question B (vs infection), using the five domains (risk of bias, inconsistency, indirectness, imprecision, publication bias); starting certainty low for observational data with possible upgrading. Supplementary File S3. |
| **RESULTS** | | | |
| **Study selection** | **16a** | Describe the results of the search and selection process, from the number of records identified in the search to the number of studies included in the review, ideally using a flow diagram. | **Reported** Sections 2.5 and 3: 299 records identified → 170 after removal of 129 duplicates → 33 sought, 28 full texts assessed → 7 studies included. PRISMA 2020 flow diagram (Figure 1). |
| **Study selection** | **16b** | Cite studies that might appear to meet the inclusion criteria, but which were excluded, and explain why they were excluded. | **Reported** Section 2.5 and Figure 1: reasons for exclusion at the full-text stage are reported by category with counts (n = 21). Every excluded report failed at least one prespecified eligibility criterion; no study that ostensibly met the inclusion criteria was excluded, so no near-miss studies required individual citation. |
| **Study characteristics** | **17** | Cite each included study and present its characteristics. | **Reported** Section 3.1 and Table 2: all 7 included studies cited with country, design, n, diagnostic criteria, age, disease phase and comparator. |
| **Risk of bias in studies** | **18** | Present assessments of risk of bias for each included study. | **Reported** Sections 2.7 and 3; Supplementary File S2 (item-level JBI appraisal and traffic-light/figure summary) and Supplementary Table S3 (per-study judgments); Supplementary File S4 (QUADAS-2 for the three diagnostic-accuracy studies). |
| **Results of individual studies** | **19** | For all outcomes, present, for each study: (a) summary statistics for each group (where appropriate) and (b) an effect estimate and its precision (e.g. confidence/credible interval), ideally using structured tables or plots. | **Reported** Sections 3.2 and 3.3 with the biomarker tables (Tables 3a–3d): full between-group versions in Supplementary Files S5; per-study summary statistics (mean ± SD / median [IQR]) by phase and comparator. Effect estimates with precision (e.g. AUCs) are presented where the primary studies reported them. |
| **Results of syntheses** | **20a** | For each synthesis, briefly summarise the characteristics and risk of bias among contributing studies. | **Reported** Sections 3.1 and 3.4 and the GRADE profile (Supplementary File S3) summarise the contributing studies and their risk of bias for each biomarker/question; QUADAS-2 risk of bias for the accuracy studies is reported in Supplementary File S4. |
| **Results of syntheses** | **20b** | Present results of all statistical syntheses conducted. If meta-analysis was done, present for each the summary estimate and its precision and measures of statistical heterogeneity. If comparing groups, describe the direction of the effect. | **Reported** Sections 3.4 (Cross-study synthesis by biomarker) and 3.5 (differentiation from other autoinflammatory diseases): direction of effect described for flare vs interval, PFAPA vs infection, and PFAPA vs SURF/FMF. Narrative synthesis; no pooled summary estimates or heterogeneity statistics (no meta-analysis). |
| **Results of syntheses** | **20c** | Present results of all investigations of possible causes of heterogeneity among study results. | **Reported** Sections 3.1 and 4: results of the heterogeneity exploration are presented narratively (e.g. the apparent ESR discrepancy explained by comparator severity; effects of assay/unit and diagnostic-criteria differences). A statistical investigation was not applicable (no meta-analysis). |
| **Results of syntheses** | **20d** | Present results of all sensitivity analyses conducted to assess the robustness of the synthesized results. | **Not applicable**  No review-level quantitative synthesis, hence no sensitivity analyses (Section 2.8). |
| **Reporting biases** | **21** | Present assessments of risk of bias due to missing results (arising from reporting biases) for each synthesis assessed. | **Reported** Supplementary File S3: publication-bias judgments are presented per biomarker within the GRADE evidence profile for each graded question. A funnel-plot analysis was not applicable (narrative synthesis). |
| **Certainty of evidence** | **22** | Present assessments of certainty (or confidence) in the body of evidence for each outcome assessed. | **Reported** Sections 3 and 3.4 and the GRADE evidence profile (Supplementary File S3): per-biomarker certainty for Questions A and B; Question C (vs other autoinflammatory disease; Section 3.5) judged very low and summarised narratively. |
| **DISCUSSION** | | | |
| **Discussion** | **23a** | Provide a general interpretation of the results in the context of other evidence. | **Reported** Section 4 (Discussion): interpretation set against mechanistic and prior literature (e.g. IFN-γ/IL-6, IP-10/CXCL10, Th1 biology). |
| **Discussion** | **23b** | Discuss any limitations of the evidence included in the review. | **Reported** Section 4: heterogeneity, small samples, incompatible units, adult comparators, non-uniform diagnostic criteria, retrospective designs, single-centre cohorts. |
| **Discussion** | **23c** | Discuss any limitations of the review processes used. | **Reported** Section 4 (no meta-analysis feasible); Section 2.2 (no PROSPERO registration); Section 2.4 (no reference-list/citation tracking or grey literature; English-language restriction). |
| **Discussion** | **23d** | Discuss implications of the results for practice, policy, and future research. | **Reported** Section 4 and Section 5 (Conclusions): use of CRP/SAA for activity, caution against use vs infection, IFN-γ/IL-6 as priority for prospective multicentre validation, head-to-head comparison with monogenic syndromes. |
| **OTHER INFORMATION** | | | |
| **Registration and protocol** | **24a** | Provide registration information for the review, including the register name and registration number, or state that the review was not registered. | **Reported** Section 2.2: the review was NOT registered (no PROSPERO/other registration); reason stated (retrospective-analytical design). |
| **Registration and protocol** | **24b** | Indicate where the review protocol can be accessed, or state that a protocol was not prepared. | **Reported** Supplementary File S1: full systematic-review protocol provided; Section 2.2 notes that selection, extraction and analysis criteria were pre-specified before data extraction (protocol prepared but not prospectively registered). |
| **Registration and protocol** | **24c** | Describe and explain any amendments to information provided at registration or in the protocol. | **Reported** Supplementary File S1, Section 6 (Deviations from Protocol): no major deviations; incorporation of the Guan et al. supplementary immune-index table into the synthesis/appraisal/GRADE is described. |
| **Support** | **25** | Describe sources of financial or non-financial support for the review, and the role of the funders or sponsors in the review. | **Reported** Section 9. Funding statement: the review received no external funding; no funder/sponsor had any role in the review. |
| **Competing interests** | **26** | Declare any competing interests of review authors. | **Reported** Section 10. Conflicts of Interest statement: the authors declare no competing interests. |
| **Availability of data, code and other materials** | **27** | Report which of the following are publicly available and where they can be found: template data collection forms; data extracted from included studies; data used for all analyses; analytic code; any other materials used in the review. | **Reported** Section 11. Data Availability Statement: all data analysed are available within the article (study-characteristics and biomarker Tables 2 and 3a–3d) and its supplementary files (Files S1–S5; Tables S1–S3); search strategies are in Supplementary Table S2. No analytic code was generated (narrative synthesis); the data-extraction template is available from the corresponding author on request. |

Abbreviations: CRP - C-reactive Protein; GRADE - Grading of Recommendations Assessment, Development and Evaluation; IFN-γ - Interferon-gamma; IL - Interleukin; JBI - Joanna Briggs Institute; NR - Not Reported;
PECO - Population–Exposure–Comparator–Outcome; PFAPA - Periodic Fever, Aphthous Stomatitis, Pharyngitis, Cervical Adenitis; PROSPERO - International Prospective Register of Systematic Reviews; SAA - Serum Amyloid A;
SURF - Syndrome of Undifferentiated Recurrent Fever; TNF-α - Tumor Necrosis Factor-Alpha; WBC - White Blood Cells.
