# Supplementary material for: Inflammatory biomarkers in pediatric PFAPA syndrome: a systematic review
Source: Front Pediatr. 2026 Sep 3;14:1934055. doi: 10.3389/fped.2026.1934055 (PMC13582404; doi:10.3389/fped.2026.1934055)
Supplement: Supplementary file 7 [file Table2.docx]

**Supplementary Table S2. The complete search strategies for PubMed/MEDLINE and Scopus.**

| **Database** | **Search Strategy** | **Results** | **Date Range** | **Last Search Date** | **Filters Applied** |
| --- | --- | --- | --- | --- | --- |
| **PubMed/MEDLINE *(Primary Search)*** | PFAPA[tiab] AND (cytokine[tiab] OR interleukin[tiab] OR CRP[tiab] OR biomarker[tiab] OR "serum amyloid"[tiab] OR calprotectin[tiab] OR interferon[tiab] OR "IL-18"[tiab] OR "inflammatory marker"[tiab]) AND (child[tiab] OR pediatric[tiab] OR children[MeSH]) | n=37 | Jan 01, 2018 – Jun 07, 2026 | Jun 07, 2026 | Not applicable |
| **PubMed/MEDLINE *(Supplementary Search — Acute-Phase Markers)*** | PFAPA[tiab] AND ("white blood cell"[tiab] OR WBC[tiab] OR "serum amyloid A"[tiab] OR calprotectin[tiab] OR "acute phase"[tiab] OR neutrophil[tiab]) | n=18 | Jan 01, 2018 – Jun 07, 2026 | Jun 07, 2026 | Not applicable |
| **PubMed/MEDLINE *(Supplementary Search — Cytokines)*** | "periodic fever" AND aphthous[tiab] AND ("IL-18"[tiab] OR "interleukin-18"[tiab] OR "interferon-gamma"[tiab] OR "IFN-gamma"[tiab] OR "tumor necrosis factor"[tiab] OR "TNF-alpha"[tiab]) | n=23 | Jan 01, 2018 – Jun 07, 2026 | Jun 07, 2026 | Not applicable |
| **Scopus *(Primary Search)*** | TITLE-ABS-KEY ( PFAPA ) AND TITLE-ABS-KEY (cytokine OR interleukin OR CRP OR biomarker OR "serum amyloid" OR calprotectin OR interferon OR "IL-18" OR "inflammatory marker") AND TITLE-ABS-KEY ( child OR pediatric OR children) | n=95 | Jan 01, 2018 – Jun 07, 2026 | Jun 07, 2026 | Not applicable |
| **Scopus *(Supplementary Search — Acute-Phase Markers)*** | TITLE-ABS-KEY ( PFAPA ) AND TITLE-ABS-KEY ("white blood cell" OR WBC OR "serum amyloid A" OR calprotectin OR "acute phase" OR neutrophil) | n=64 | Jan 01, 2018 – Jun 07, 2026 | Jun 07, 2026 | Not applicable |
| **Scopus *(Supplementary Search — Cytokines)*** | TITLE-ABS-KEY ( "periodic fever" ) AND TITLE-ABS-KEY ( aphthous ) AND TITLE-ABS-KEY ("IL-18" OR "interleukin-18" OR "interferon-gamma" OR "IFN-gamma" OR "tumor necrosis factor" OR "TNF-alpha") | n=62 | Jan 01, 2018 – Jun 07, 2026 | Jun 07, 2026 | Not applicable |

*Note: PubMed/MEDLINE and Scopus were selected because, together, they provide broad coverage of the pediatric rheumatology and immunology literature relevant to PFAPA. Embase was not searched because the review team lacked institutional access. This restriction is discussed as a limitation in the main text (Discussion, Section 4).*
