# Supplementary material for: Inflammatory biomarkers in pediatric PFAPA syndrome: a systematic review
Source: Front Pediatr. 2026 Sep 3;14:1934055. doi: 10.3389/fped.2026.1934055 (PMC13582404; doi:10.3389/fped.2026.1934055)
Supplement: Supplementary file 8 [file Table3.docx]

**Supplementary Table S3. Joanna Briggs Institute (JBI) Risk-of-Bias Appraisal for the 7 Included Studies**

| **Author (Year)** | **Study design** | **JBI overall rating** | **Key bias domains flagged** |
| --- | --- | --- | --- |
| **Guan et al., 2026 [1]** | Retrospective cohort (10-year, single-centre) | **Moderate** | Tertiary-referral selection bias; infectious and SURF comparators present (Guan et al.’s Supplementary Tables S1–S2) but neither age-matched nor adjusted; cytokines reported as IQR without medians; no blinding |
| **Zhu et al., 2026 [2]** | Retrospective single-centre (PFAPA vs bacterial infection) | **Low to Moderate** | Retrospective single-centre design; residual confounding by sampling time (2 vs 6 days) despite a sensitivity analysis; no inter-episode data; no blinding |
| **Kapustova et al., 2023 [3]** | Prospective cohort (paired flare vs interval) | **Low** | No external (infectious or autoinflammatory) comparator; less-validated serum calprotectin assay; unbalanced ketotifen allocation; no blinding |
| **Onur & Onur, 2023 [4]** | Prospective observational (PFAPA vs streptococcal pharyngitis) | **Moderate** | Age mismatch between groups not adjusted (age strongest discriminating variable, AUC 0.713); probable unacknowledged CRP unit error (mg/dL vs mg/L); no cytokines/SAA; no blinding |
| **Lazea et al., 2022 [5]** | Retrospective single-centre cohort (paired episode vs inter-episode) | **Moderate to High** | Small sample (n=39); no genetic exclusion of monogenic mimics; implausible inter-episode WBC range (likely transcription error); cytokines/SAA not measured; no blinding |
| **Nakano et al., 2021 [6]** | Retrospective comparative (PFAPA vs recurrent tonsillitis) | **High** | Critically small sample (n=3 vs 4); marked unadjusted age imbalance (5.2 vs 11.5 yr); IFN-γ in incompatible units (IU/mL); negligible statistical power; no blinding |
| **Takeuchi et al., 2019 [7]** | Retrospective cohort (18-year; vs infection, FMF, healthy) | **Low to Moderate** | Healthy comparators were adults, not age-matched children (indirectness); cytokine subset (n=94); skewed cytokine data summarised as mean ± SD (SD > mean); no blinding |

*Appraisal performed with the JBI Critical Appraisal Checklist for Analytical Cross-Sectional Studies, applied uniformly to all seven cohorts; for the two paired flare-versus-interval studies (Kapustova, Lazea), the equivalent JBI cohort items were additionally considered. Item-level scoring and the traffic-light summary are presented in Supplementary File S2, available with the main text. A separate QUADAS-2 appraisal of the three diagnostic-accuracy studies (Onur & Onur, Zhu et al., Takeuchi et al.) is provided in Supplementary File S4.*

References:

1.	Guan, C.; Zhou, Q.; Sun, J.; Ying, W.; Hui, X.; Hou, J.; Liu, L.; Wang, C.; Liu, L.; Sun, B.; et al. The Etiological Spectrum of Pediatric FUO and Clinical Management of PFAPA/SURF: A Ten-Year Retrospective Study. Front. Immunol. **2026**, 17, doi:10.3389/fimmu.2026.1791904.

2.	Zhu, X.; Yang, Z.; Huang, Y.; Luo, Y.; Yang, J.; He, T. Cytokine Profiles and Laboratory Parameters as Indicators to Distinguish Children with PFAPA and Bacterial Infection. Front. Immunol. **2026**, 17, doi:10.3389/fimmu.2026.1683221.

3.	Kapustova, L.; Banovcin, P.; Bobcakova, A.; Jurkova Malicherova, E.; Kapustova, D.; Petrovicova, O.; Slenker, B.; Markocsy, A.; Oleksak, F.; Vorcakova, K.; et al. The Use of Ketotifen as Long-Term Anti-Inflammatory Prophylaxis in Children with PFAPA Syndrome. Front Immunol **2023**, 14, 1302875, doi:10.3389/fimmu.2023.1302875.

4.	Onur, H.; Onur, A.R. Diagnostic Performance of Routine Blood Parameters in Periodic Fever, Aphthous Stomatitis, Pharyngitis, and Adenitis Syndrome. Journal of Clinical Laboratory Analysis **2023**, 37, e24934, doi:10.1002/jcla.24934.

5.	Lazea, C.; Damian, L.; Vulturar, R.; Lazar, C. PFAPA Syndrome: Clinical, Laboratory and Therapeutic Features in a Single-Centre Cohort. Int J Gen Med **2022**, 15, 6871–6880, doi:10.2147/IJGM.S373942.

6.	Nakano, S.; Kondo, E.; Iwasaki, H.; Akizuki, H.; Matsuda, K.; Azuma, T.; Sato, G.; Kitamura, Y.; Abe, K.; Takeda, N. Differential Cytokine Profiles in Pediatric Patients with PFAPA Syndrome and Recurrent Tonsillitis. J Med Invest **2021**, 68, 38–41, doi:10.2152/jmi.68.38.

7.	Takeuchi, Y.; Shigemura, T.; Kobayashi, N.; Nagumo, H.; Furumoto, M.; Ogasawara, K.; Fujii, H.; Takizawa, M.; Soga, T.; Matoba, H.; et al. Clinical Features and New Diagnostic Criteria for the Syndrome of Periodic Fever, Aphthous Stomatitis, Pharyngitis, and Cervical Adenitis. International Journal of Rheumatic Diseases **2019**, 22, 1489–1497, doi:10.1111/1756-185X.13610.
